# The changing landscape of immune cells in the fetal mouse testis

**DOI:** 10.1007/s00418-022-02129-6

**Published:** 2022-07-12

**Authors:** Samira Hosseini, Sarah C. Moody, Daniela Fietz, Sivanjah Indumathy, Hans-Christian Schuppe, Mark P. Hedger, Kate L. Loveland

**Affiliations:** 1 Centre for Reproductive Health, Hudson Institute of Medical Research, 27-31 Wright St, Clayton, VIC 3168 Australia; 2 Department of Molecular and Translational Sciences, Monash University, Clayton, VIC Australia; 3 Institute of Veterinary Anatomy, Histology and Embryology, Justus Liebig University Giessen, Giessen, Germany; 4 Department of Urology, Pediatric Urology and Andrology, Justus Liebig University Giessen, Giessen, Germany; 5 Department of Anatomy and Developmental Biology, School of Biomedical Sciences, Monash University, Clayton, VIC Australia

**Keywords:** Fetal testis development, Macrophages, T cells, Granulocytes, Immune cell localisation, Male germ cells

## Abstract

Fetal testis growth involves cell influx and extensive remodeling. Immediately after sex determination in mouse, macrophages enable normal cord formation and removal of inappropriately positioned cells. This study provides new information about macrophages and other immune cells after cord formation in fetal testes, including their density, distribution, and close cellular contacts. C57BL6J mouse testes from embryonic day (E) 13.5 to birth (post-natal day 0; PND0), were examined using immunofluorescence, immunohistochemistry, and RT-qPCR to identify macrophages (F4/80, CD206, MHCII), T cells (CD3), granulocytes/neutrophils (Ly6G), and germ cells (DDX4). F4/80^+^ cells were the most abundant, comprising 90% of CD45^+^ cells at E13.5 and declining to 65% at PND0. Changes in size, shape, and markers (CD206 and MHCII) documented during this interval align with the understanding that F4/80^+^ cells have different origins during embryonic life. CD3^+^ cells and F4/80^−^/MHCII^+^ were absent to rare until PND0. Ly6G^+^ cells were scarce at E13.5 but increased robustly by PND0 to represent half of the CD45^+^ cells. These immunofluorescence data were in accord with transcript analysis, which showed that immune marker mRNAs increased with testis age. F4/80^+^ and Ly6G^+^ cells were frequently inside cords adjacent to germ cells at E13.5 and E15.5. F4/80^+^ cells were often in clusters next to other immune cells. Macrophages inside cords at E13.5 and E15.5 (F4/80^Hi^/CD206^+^) were different from macrophages at PND0 (F4/80^Dim^/CD206^−^), indicating that they have distinct origins. This histological quantification coupled with transcript information identifies new cellular interactions for immune cells in fetal testis morphogenesis, and highlights new avenues for studies of their functional significance.

**Supplementary Information:**

The online version contains supplementary material available at 10.1007/s00418-022-02129-6.

## Introduction

The mouse testis first forms in fetal life, with the assignment of male fate enabled by expression of *SRY* at embryonic day (E) 10.5 in Sertoli cells (Koopman et al. 1991). This initiates the emergence of the somatic lineages central to adult testis function, and commits the sexually indifferent primordial germ cells to the male fate by E12.5, when they become gonocytes (Adams et al. 2002). The fetal testis then undergoes major cellular and structural transformations, which enable it to ultimately perform two key functions in adult life: hormone secretion and production of spermatozoa (Wilhelm et al. 2007, 2013). There is increasing interest in understanding the distribution and roles of immune cells during the dynamic remodeling that characterises fetal and newborn testis development, particularly because processes that disrupt normal development may increase the risk of adult infertility and testicular neoplasia, including testicular germ cell tumors arising from germ cell neoplasia in situ (Skakkebaek et al. 2016). Macrophages have been of particular interest because cells in the myeloid lineage perform key roles in tissue remodeling and homeostasis. They are the most abundant immune cells in the testis at all ages (Mossadegh-Keller and Sieweke, 2018). Macrophage depletion during embryonic life has demonstrated that testicular macrophages regulate testicular vascularisation and morphogenesis in the mouse (DeFalco et al. 2014). After birth, testicular macrophages play key roles in homeostasis, supporting spermatogonial differentiation, including through the production of CSF-1 and the promotion of Leydig cell testosterone production (DeFalco et al. 2015; Fijak et al. 2017, 2018; Mossadegh-Keller and Sieweke, 2018).

In the normal adult testis, resident macrophages are classified into two major groups: interstitial macrophages that are in close contact with Leydig cells within the interstitium, and peritubular macrophages on the seminiferous tubule surface, close to peritubular myoid cells. These are each predicted to perform different functions, based on their locations and distinct expression profiles of functional markers (DeFalco et al. 2015; Mossadegh-Keller et al. 2017; Lokka et al. 2020; Indumathy et al. 2020; Wang et al. 2021). Recent investigations have defined the origins of testicular macrophages using fate mapping and single-cell RNA-sequencing (scRNAseq) analyses. The first study using fate-mapping to examine testicular macrophage emergence at the earliest stages of fetal testis development provided evidence that macrophages present in the adult testis arise from the yolk sac (DeFalco et al. 2014). A subsequent analysis demonstrated that adult interstitial macrophages are yolk sac-derived, while peritubular macrophages emerge exclusively after birth and are bone marrow-derived (Mossadegh-Keller et al. 2017). More recent studies incorporating scRNAseq revealed that interstitial macrophages mainly originate from fetal liver-derived precursors, while peritubular macrophages develop prenatally and are detectable at birth (Lokka et al. 2020; Wang et al. 2021). The requirement of macrophages for normal testis cord formation during early fetal life was demonstrated by the outcome of their selective depletion. In addition, macrophage engulfment of germ cells and Sertoli cells outside of nascent cords was identified at E11.5 and E12.5 (DeFalco et al. 2014).

CD206, also known as mannose receptor-1 (MRC-1), is present on both immune and non-immune cells (Sheikh et al. 2000), and is used as a key marker of macrophages with an anti-inflammatory and tolerogenic phenotype. In contrast, a high level of the MHC class II molecule (MHCII) is critical for macrophage initiation of antigen-specific immune responses that can lead to inflammation (Shapouri‐Moghaddam et al. 2018). In the postnatal testis, CD206 is expressed on interstitial macrophages and MHCII is exclusively expressed on peritubular macrophages (Mossadegh-Keller et al. 2017). A proposed function for MHCII on peritubular testicular macrophages is to present spermatid-derived antigens to regulatory T cells (Treg), thereby inducing cell-dependent physiological tolerance against these antigens (Smith et al. 2016; Tung et al. 2017; Mossadegh-Keller and Sieweke, 2018; Heinrich and DeFalco, 2020). Although there are conflicting reports regarding the detection of peritubular macrophages at birth (Lokka et al. 2020; Wang et al. 2021), we hypothesized that, in fetal and newborn mouse testis, the dominant testicular macrophage population would be CD206^+^ MHCII^−^ in order to sustain an immune regulatory and tolerogenic microenvironment during testis remodeling and morphogenesis.

We were intrigued by the possibility that additional myeloid and lymphoid cells could be integral to early testis development. Based on the known functional relationships between T cells, neutrophils, and macrophages during inflammation, we investigated whether T cells and neutrophils could also be present in the fetal testis. T cells (CD3^+^) can have either inflammatory or immune tolerogenic functions, depending on environmental cues (Khan and Ghazanfar et al. 2018). They are relatively common in the adult testis, representing approximately 25% of all immune cells in the adult rat testis (Bhushan et al. 2020). Testicular macrophages, acting as antigen-presenting cells, can trigger adaptive immunity, auto-immunity, and inflammation in the testis; they can also present germ cell-derived antigens to the T cells under regulatory conditions to convert them to Treg (Bhushan et al. 2020; Wang et al. 2021), but information about these cells in the fetal testis is lacking.

Best understood as the first line of defense against pathogens, key cells in the granulocyte lineage include neutrophils, basophils, and eosinophils as the most abundant leucocytes, that are generally best known for their role in inducing inflammatory factors (Lin and Loré 2017). Their engagement may ultimately cause host tissue damage and increase both disease pathogenesis and severity if not cleared by the macrophages, which typically restore homeostasis (Kobayashi and DeLeo 2009; Soehnlein and Lindbom 2010). In adults, neutrophils are abundant in circulation, and are relatively short-lived, highly migratory cells which contribute to organ homeostasis, exhibiting daily movement in and out of organs in mice (Kobayashi and DeLeo, 2009; Casanova-Acebes et al. 2013, Lok et al. 2019; Lin and Loré 2017). In contrast, neutrophil roles in healthy organ development are not established. Most studies of testicular neutrophils have focused on the adult in rats (Lysiak et al. 2001; Sukhotnik et al. 2007; Arena et al. 2020) and humans (Yamada et al. 2016; Bolat et al. 2017), but their recruitment in the fetal mouse testis has been demonstrated at E12.5 in association with vasculature development (DeFalco et al. 2014). Neutrophils have been documented in the human testis in conditions of acute bacterial epididymo-orchitis (Schuppe and Bergmann 2013; Fijak et al. 2018), and their presence in relatively high density in certain testicular germ cell tumors was proposed as a survival prognostic factor (Yamada et al. 2016). Beyond this, the presence of granulocytes, including neutrophils, and their function during fetal testis development in the mouse, rat, and human are undocumented.

This study addresses the emergence and localization of macrophages and granulocytes, which arise from a common myeloid progenitor cell (Rosmarin et al. 2005) and CD3^+^ cells, during murine in utero development between E13.5 to birth (post-natal day 0; PND0). The frequency, localization, and close cell contacts of macrophages (F4/80^+^ cells), T cells (CD3^+^ cells), and neutrophils (Ly6G^+^ cells) were documented following immunofluorescence (IF) and immunohistochemistry (IHC), including in relationship to gonocytes (DDX4^+^ cells). This histological approach avoids the potential for under-reporting of cell types that can arise following tissue-dissociation for flow cytometry (Steinert et al 2015), and it was extended by monitoring changes in the levels of transcripts relating to each of these cell types. The outcomes provide new ideas about what immune cells may contribute to normal mouse testis development to set the stage for adult fertility.

## Materials and methods

### Animals

Mice were housed at the specific pathogen-free (SPF) Monash Animal Research Platform or at the SPF Monash Monash Medical Centre Animal Facility in accordance with the Australian Code of Practice for the Care and Use of Animals for Scientific Purposes, with a 12-h light and 12-h dark cycle, with food and water available ad libitum. All investigations conformed to the National Health and Medical Research Council/Commonwealth Scientific and Industrial Research Organisation/Animal Advisory Committee Code of Practice for Care and Use of Animals for Experimental Purposes, and were approved by the Monash University Standing Committee on Ethics in Animal Experimentation. Timed matings of C57BL/6 J mice were performed, with the day of plug identified as embryonic day (E) 0.5. Pregnant mice were killed by cervical dislocation, and the embryos (at E13.5, E15.5) were immediately removed from the uterus, killed, and the testes collected. Embryonic age was verified using forelimb and hindlimb development stages. At least four pups per age (at E13.5, E15.5, and PND0) were analyzed from more than one litter for each age. Testes from three individual C57BL/6 J mice were examined on PNDs 2, 7, 10, 15, 20, and adult to visualize Ly6G^+^ cell distribution.

### Tissue preparation

Two different fixation and embedding approaches were employed. For IHC experiments, one testis from each mouse was immersed at room temperature (RT) in Bouin’s solution (30 min for E13.5, 60 min for E15.5, and 90 min for PND0), followed by washing in 70% ethanol (3 × 1 h). Fixed tissues were processed, embedded in paraffin, and sequential sections of 5 μm thickness were collected onto Superfrost Plus microscope slides (Thermo Fisher Scientific, Waltham, MA, USA). For IF analyses, one testis from each mouse was fixed in 4% paraformaldehyde overnight at 4 °C, then cryoprotected in phosphate-buffered saline (PBS) first with 15% (w/v) sucrose overnight at 4 °C, then with 30% sucrose solution the following day. Tissues were embedded in Tissue Tek OCT compound (Sakura Finetek, USA) then stored at − 80 °C. Cryosections (5 μm) were placed onto Superfrost Plus microscope slides for immediate use or stored at − 80 °C.

Each testis was entirely serially sectioned, with three sections placed on each slide. Four slides corresponding to the centre of the testis were examined with each antibody, ensuring that replicate sections for each antibody were spaced at 15–30 µm to prevent double-counting of individual cells. Section cross-sectional area was measured using ImageJ software (v.2.1.0; National Institutes of Health, Bethesda, MD, USA,); this did not differ significantly between samples within each age group (Supplementary Fig. S1A). Tissue embedding and sectioning were performed by staff at the Monash Health Research Precinct node of the Monash Histology Platform, Monash University.

For RNA extraction, testes at each age (*n* = 3/age) were dissected away from the mesonephros, snap-frozen immediately on dry ice, and stored at – 80 °C.

### Immunostaining

Immunostaining was performed using antibodies (detailed in Supplementary Table S1, including citations of prior usage) to detect all immune cells (anti-CD45), macrophages (anti-F4/80, anti-CD206, anti-MHC Class II), T cells (anti-CD3), neutrophils (anti-Ly6G), laminin (anti-laminin), and germ cells (anti-DDX4). Adult mouse spleen was used as a positive control for antibodies to immune cell markers, and adult and newborn mouse testis were used for antibodies to laminin and germ cells. Each directly conjugated antibody bound as expected to cells in appropriate locations and with appropriate morphology (e.g., size, nuclear shape). Negative controls for unconjugated primary antibodies lacked primary antibodies and were used to establish background binding of secondary antibodies.

For IHC, sections were deparaffinized and rehydrated through a series of 5-min washes: twice each in histolene (Trajan, Australia) and 100% ethanol, then once each in 95, 80, 70, 50, and 30% ethanol and deionised water. Heat‐induced antigen retrieval was performed by immersing slides in Tris–EDTA buffer (10 mM Tris Base, 1 mM EDTA, 0.05% Triton X-100 (Sigma‐Aldrich), pH 9.0); the container with slides was microwaved for 4–5 min at 800 W, then 9 min at 450 W, followed by cooling at RT for 30 min. Sections were incubated for 20 min at RT with 3% hydrogen peroxide (Merck Millipore) to quench endogenous peroxidase, followed by washes with Tris-buffered saline (TBS; 50 mM Tris–Cl, 150 mM NaCl, pH 7.5; 3 × 5 min). Sections were next incubated in blocking solution, consisting of 5% normal serum from the animal, in which the secondary antibody was raised (5% in TBS) in a humid chamber at RT for 1 h. Primary antibodies diluted in TBS with 1% bovine serum albumin (BSA; Sigma-Aldrich) were applied overnight at 4 °C.

Slides were washed three times, 3 min each, between incubations at RT using TBS. Controls for non‐specific secondary antibody binding in all experiments lacked primary antibody; these consistently showed no signal. Primary antibody binding was detected using a biotinylated secondary antibody in a humid chamber for 1 h at RT. After consecutive washes (3 × 3 min), Vectastain Elite ABC kit reagents were added (Vector Laboratories, USA) according to the manufacturer’s instructions. Antibody binding was detected as a brown precipitate following development with 3, 3‐diaminobenzidine tetrahydrochloride (Agilent Technologies, USA), then sections were counterstained with Harris hematoxylin (Sigma-Aldrich). Stained slides were then dehydrated through a graded ethanol series (once each in 30, 50, 70, 80, and 95% ethanol for 3 min), plus twice in 100% ethanol and histosol (5 min). Finally, the slides were mounted under glass coverslips using DPX mounting media (Sigma‐Aldrich) and left flat overnight to air-dry before imaging.

Slides for indirect and direct IF stored at − 80 °C were air-dried for 20 min at RT and rehydrated in PBS for 10 min. To permeabilize the sections, 0.1% Triton X-100 (Sigma-Aldrich) in PBS was applied for 5 min at RT. Slides were washed in PBS (×3), followed by a two-step blocking protocol in a humid chamber at RT. First, 5% Mouse on Mouse Blocking Reagent (Vector Laboratories) diluted in TBS was applied to sections for 30 min, then the slides were washed in PBS (3 min), and, finally, sections were exposed to 5% normal serum (from the species of secondary antibody origin)/2% BSA/PBS for 1 h. Blocking solution was replaced with unconjugated primary antibodies (for indirect IF) diluted in 2% BSA/0.1% Triton X-100/PBS, and the slides were incubated overnight in a humid chamber at 4 °C. Negative control sections lacked primary antibody. The next day, slides were washed once in 0.1% Triton X-100/PBS (3 min), twice in PBS (3 min each), and appropriate conjugated secondary antibodies were applied for 1 h at RT, with the slides protected from light from this point onwards. Secondary antibodies were removed, and the slides were washed as above. For direct immunofluorescence, after the blocking step, conjugated primary antibodies were added overnight at 4 °C, the slides were washed as above, and the sections were mounted under a glass coverslip using ProLong Gold Mountant (Invitrogen, USA) and stored at 4 °C until scanning for image analysis (within 24 h).

### Imaging and morphometric analysis

Immunostained sections were scanned using an Olympus VS120 Slide Scanner (16-bit Fluorescence; Olympus XM10 Monochrome Camera) and analyzed using OlyVIA Software (2.9.1 Viewer; Olympus Life Science). The minimum detection level at which no fluorescence could be seen on the negative control was established as the baseline, and signals visible above this were considered genuine; detection levels were set identically for each section treated with the same primary and secondary antibody in an individual experiment to ensure consistency. Cells with a distinct signal evident at 6000 or higher on the intensity scale were labeled as ‘Hi’, cells with a signal between 3000 and 6000 were identified as ‘Int’, and a signal less than 3000 was recorded as ‘Dim’.

Immune cells were identified based on the presence of a well-defined nucleus visualized using DAPI (4′,6-diamidino-2-phenylindole; Invitrogen, D3571, 1:1000 in PBS). The position of each positive macrophage within an individual section was scored as being in the ‘perimeter’ area (the region between the capsule and cords) or ‘interior’ area (inside cords and interstitium) (Supplementary data S1B, S1C). Macrophage density was reported by adding the number of macrophages detected in the perimeter and interior areas of a section, divided by the section cross-sectional area (µm)^2^, measured using ImageJ software. Analysis was initially performed by blinded counting of three slides by two independent operators, with a variation of 5.3% for total macrophage numbers. The entire series was subsequently counted blinded, by a single operator.

### RNA extraction, cDNA synthesis and quantitative RT-PCR (qRT-PCR)

Transcripts were measured by qRT-PCR in C57BL6J mouse testes at E13.5, E15.5, and PND0, from 3 independent samples per age. RNA extraction and DNase treatment using the RNeasy Mini Kit (Qiagen, Germany) was performed according to manufacturer's instructions. RNA purity and concentrations were quantified on a NanoDrop™ OneC (Thermo Scientific, USA). Complementary DNA (cDNA) was synthesized from 100 ng RNA with 500 ng oligo dTs (Promega, USA), 50 ng random primers (Promega), and 200 units of SuperScript III Reverse Transcriptase (Thermo Fisher Scientific) per sample, according to the enzyme manufacturer’s protocol. Primers designed to span exon–exon junctions in target genes (Integrated DNA Technologies, USA) are listed in Supplementary Table S2. qRT-PCR was performed using SYBR-green and specific primer pairs (total 10 ng) measuring each sample in triplicate; negative controls lacking Superscript III (− RT) were included for each sample. Runs conducted on Applied Biosystems QuantStudio 6 (Thermo Fisher Scientific) in the Genomics Centre, Monash Health Translation Precinct, Clayton, AU) used the following cycling conditions: denaturation at 95 °C for 10 min, followed by amplification for 40 cycles of 95 °C for 30 s, 62 °C for 30 s, and 72 °C for 30 s.

The RPLP0 housekeeping gene transcript was used to normalize transcript loading. Data were analyzed using the standard curve method generated by 6 serial dilutions with 3 technical replicates using QuantStudio 6 software (v.1.3; ThermoFisher Scientific). Relative quantification of each transcript is presented as the quantity mean level. A single amplification product was detected for each target.

### Statistical analysis

GraphPad Software (v.9.1.2; San Diego, USA) was used for statistical analysis and graphing. A one-way ANOVA and Tukey’s multiple comparison post hoc and unpaired *t* test with Welch’s correction were employed to analyze differences between age groups, as indicated. Values were considered significantly different if *p* < 0.05.

## Results

**Fig. 1:**
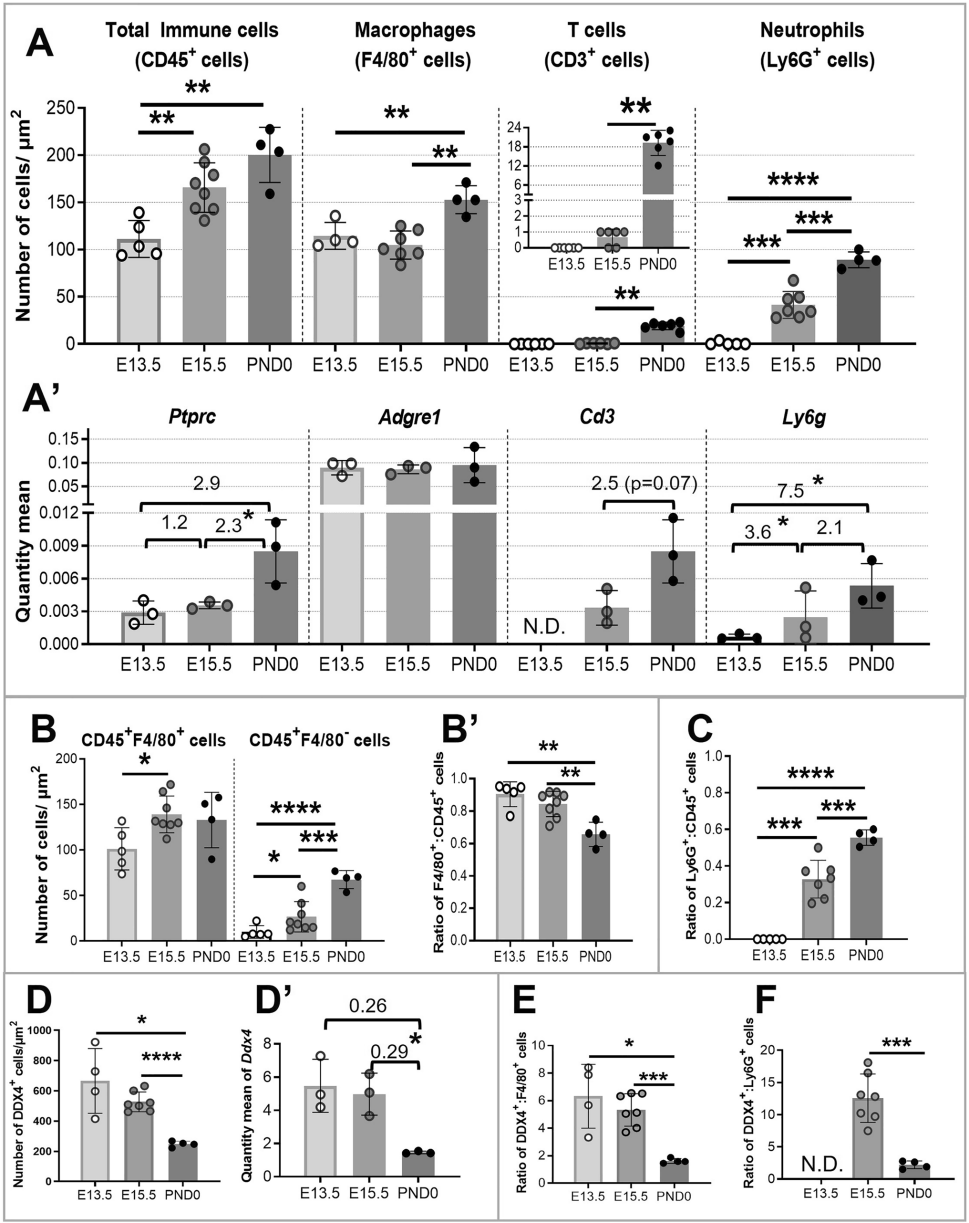
Quantitative changes in immune cell populations in fetal testes, E13.5 to PND0. **A** The density of immune cells (CD45^+^ cells), macrophages (F4/80^+^ cells), T cells (CD3^+^ cells), and neutrophils (Ly6G^+^ cells) in E13.5, E15.5, and PND0 testes determined by counting all cells in sections labeled using immunofluorescence. **A′** RT-qPCR results reported as quantity mean of transcripts encoding corresponding immune cell markers normalized to RPLP0, with fold-change values between age groups indicated. *N.D.* Not detected. **B** The density of CD45^+^/F4/80^+^ (macrophages) and CD45^+^/F4/80^−^ cells (non-macrophages). **B′** The ratio of F4/80^+^:CD45^+^ cells. **C** The ratio of Ly6G^+^:CD45^+^ cells. *D* The density of germ cells (DDX4^+^ cells) from E13.5 to PND 0. **D′** qRT-PCR quantity mean of transcripts encoding germ cell marker *Ddx4* normalized to *Rplp0*, with fold-change values between age groups indicated. **E** The ratio of DDX4^+^ to F4/80^+^. **F** The ratio of DDX4^+^ to Ly6G^+^ cells (neutrophils). *Circles* show results from individual animals; *values* represent mean ± SD. Significance determined using 2-tailed unpaired *t* test. *p ≤ 0.05 , **p ≤ 0.01 , ***p ≤ 0.001 , ****p ≤ 0.0001

Analysis of immunofluorescence was conducted to enumerate and to categorize the localisation of the total immune cell population (identified as CD45^+^ cells), macrophages (F4/80^+^ cells), T cells (CD3^+^ cells), and granulocytes (Ly6G^+^ cells), including in relationship to male germ cells (DDX4^+^ cells) in sections of E13.5 and E15.5 and newborn mouse testes (PND0) (Fig. 1). A small proportion of Ly6G^+^ cells also displayed a F4/80^Dim^ signal.

Testicular F4/80^+^ cells exhibited two distinct shapes (Supplementary Figure S2). The predominant macrophage population at E13.5 consisted of relatively large and elongated cells, greater than 10 µm in diameter and featuring a round nucleus and intense F4/80 signal (F4/80^Hi^, defined as visible at Fixed Scaling of 6000 in OlyVIA Software). A population of smaller (< 10 µm) macrophages displayed a lesser F4/80 signal; these were first detected at E15.5, and consisted of rounded cells with either a rounded or polymorphic nucleus (Supplementary Figure S2). We speculated that these small, rounded F4/80^Dim^ cells with either a band-shaped or segmented nucleus could be granulocytes.

### The number of testicular immune cells increases significantly during fetal development

The density of each immune cell type per testis cross-section increased significantly as the testis grew, indicating a numerical increase over time (Fig. 1A). CD45^+^ cell density increased 1.74-fold from E13.5 to PND0, while F4/80^+^ cells increased 1.34-fold, and Ly6G^+^ cells a remarkable 114-fold. No CD3^+^ cells (T cells) were detected in the E13.5 testis; they were rare at E15.5 (0–1/section), and more frequent at PND0 (10–20/section) (Supplementary Figure S3). This matched the profile of the *Cd3* transcript (Fig. 1A’), which was barely detectable at E13.5, but increased 2.5-fold from E15.5 to PND0. Transcript levels reinforced the immunofluorescence data, showing the same trend for *Ptprc* (encoding CD45), *Cd3* (encoding CD3), and *Ly6g* (encoding Ly6G) over this period of testis development (Fig. 1A′). As stated earlier, the macrophage population transitioned from large, elongated F4/80^Hi^ cells at E13.5, to include both F4/80^Hi^ and small rounded F4/80^Dim^ macrophages at E15.5 and PND0 (Supplementary Fig. S2). This difference in the F4/80 marker level was aligned with qRT-PCR data; despite a significant increase in macrophage number from E13.5 to PND0, the *Adgre1* transcript level (encoding F4/80) did not change (Fig. 1A′). The proportion of macrophages relative to the total immune cell number (the F4/80^+^:CD45^+^ cell ratio) significantly decreased: from 90% at E13.5, to 84% at E15.5, and to 65% at PND0 (Fig. 1B, B′). On the other hand, between E15.5 to PND0, there was a massive numerical increase in other immune cell types. Detection of Ly6G^+^ cells revealed that more than half (55%) of CD45^+^ cells in the newborn testis were granulocytes (Fig. 1C), and, as discussed below, were, based on their appearance, most likely neutrophils. The overlap between CD45^+^/F4/80^+^ cells (65%) and CD45^+^/Ly6G^+^ cells (55%) could be explained by a small proportion of small, rounded CD45^+^ cells which expressed both F4/80 and Ly6G markers.

### Changing ratios between germ and immune cell populations

Both the density of DDX4^+^ cells (germ cells) and the levels of the *Ddx4* transcript decreased from E13.5 to PND0 (Fig. 1D, D´). The decreasing ratio of DDX4^+^:F4/80^+^ cells reflects the increasing density of macrophages and the decreasing density of germ cells during this interval (Fig. 1E). The transiently elevated ratio of DDX4^+^:Ly6G^+^ cells at E15.5 arises from the inverse relationship in the density of each population during this interval (Fig. 1F). Since neutrophils are absent to rare at E13.5, no calculation was performed at this timepoint.

### A marked redistribution of macrophages from the testis perimeter to interior

The distribution of testicular macrophages changed markedly from E13.5 to PND0, from the perimeter into the interior of each testis section. At E13.5, 90% of F4/80^+^ cells were in the perimeter region, compared to only 26% by PND0 (Table 1; Supplementary Figure S4).

**Table 1:** Quantification of macrophage distribution in fetal and newborn testis sections

	E13.5 (*n* = 5)	E15.5 (*n* = 8)	PND0 (*n* = 4)
Perimeter			
Average mean (min–max)	16 (6–23)	23.13 (12–33)	36.25 (25–50)
Interior			
Mean (min–max)	3.6 (6–18)	27.25 (20–39)	101.3 (85–118)
Ratio of P:I	4.4	0.84	0.35
Total number (P + I)			
Mean ± standard deviation (min–max)	19.6 ± 6.5 (20–37)	50.38 ± 6.7 (42–59)	137.5 ± 8.7 (112–152)

For each individual animal, four sections were examined from the middle of each testis. The perimeter (**P**) region of each section is delineated as the area lacking cords, in contrast to the interior (*I*) region (illustrated in Supplementary Fig. 1)

*n* number of animals in each group

**Fig. 2:**
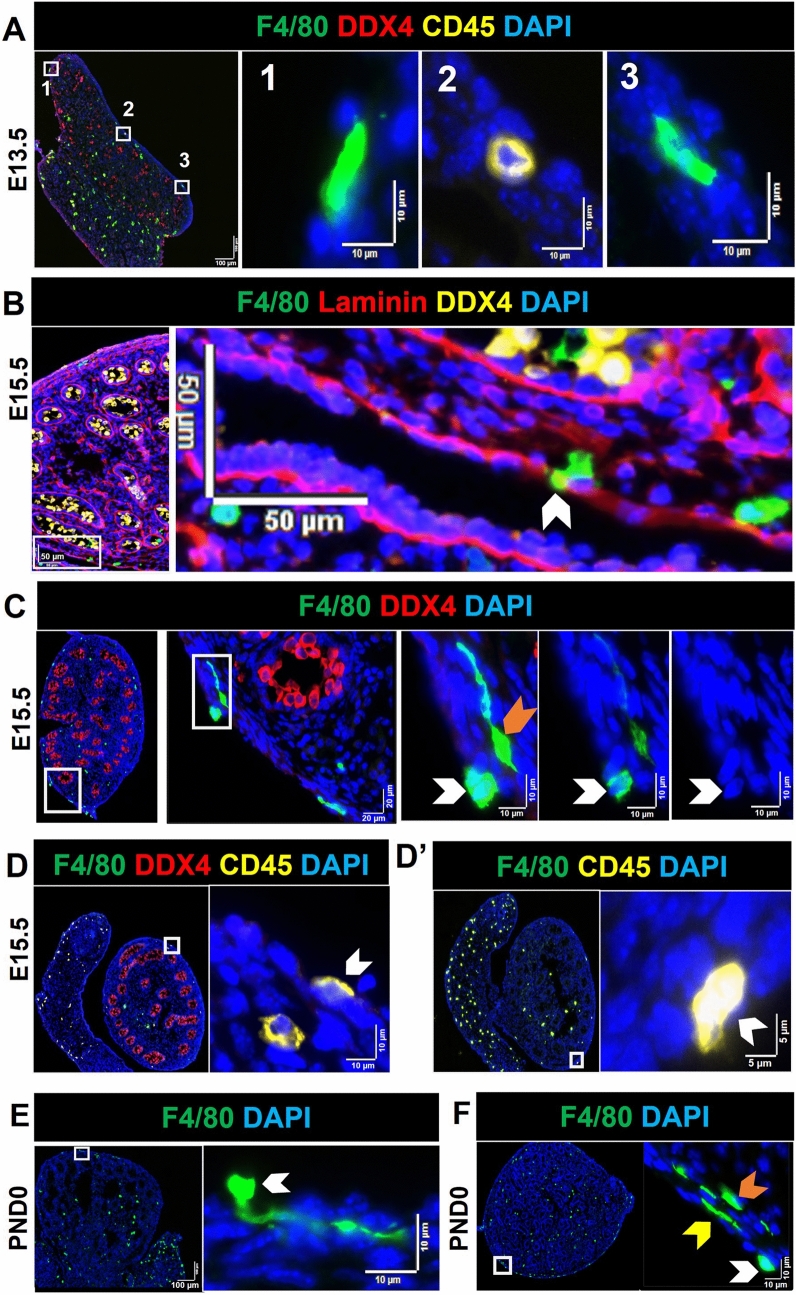
Immune cells attached to the outer layer of the testis capsule and amongst the mesothelial cells of the coelomic epithelium (in the perimeter region), visualized by immunofluorescence. Lower magnification images on *left*, with *white boxes* indicating regions shown in high magnification on *right hand panels*; dimensions as indicated. **A** Whole E13.5 testis Sect. (**1**) F4/80^+^ cell attached to testis capsule outer layer. (**2**) CD45^+^/F4/80^−^ cell amongst mesothelial cells. (**3**) F4/80^+^ cell inside the capsule, amongst mesothelial cells. **B** F4/80^+^ cell embedded in the capsule lamina (*white arrow*). **C** F4/80^+^ cells attached to the capsule outer layer (*white arrows*) and in the perimeter (*orange arrow*). **D** and **D′** CD45^+^/F4/80^−^ cells attached to the outer layer of capsule (*white arrows*). **E** In PND0 testis section, F4/80^+^ cell attached to the capsule outer layer. **F** At PND0, F4/80^+^ cells attached to the outer capsule layer (*white arrow*), amongst mesothelial cells (*yellow arrow*), and in perimeter area (*orange arrow*). *F4/80* pan-macrophage marker, *CD45* pan-immune cell marker, *DDX4* pan-germ cell marker, *laminin* extracellular matrix, *DAPI* nuclei. *Marker colors* are indicated above each panel set

Macrophages were also frequently observed attached to the outer layer of the testis capsule or among the mesothelial cells of the coelomic epithelium in the perimeter area; this was particularly evident at E13.5 and E15.5 (Fig. 2).

### Co-localization of immune cells and germ cells

**Fig. 3:**
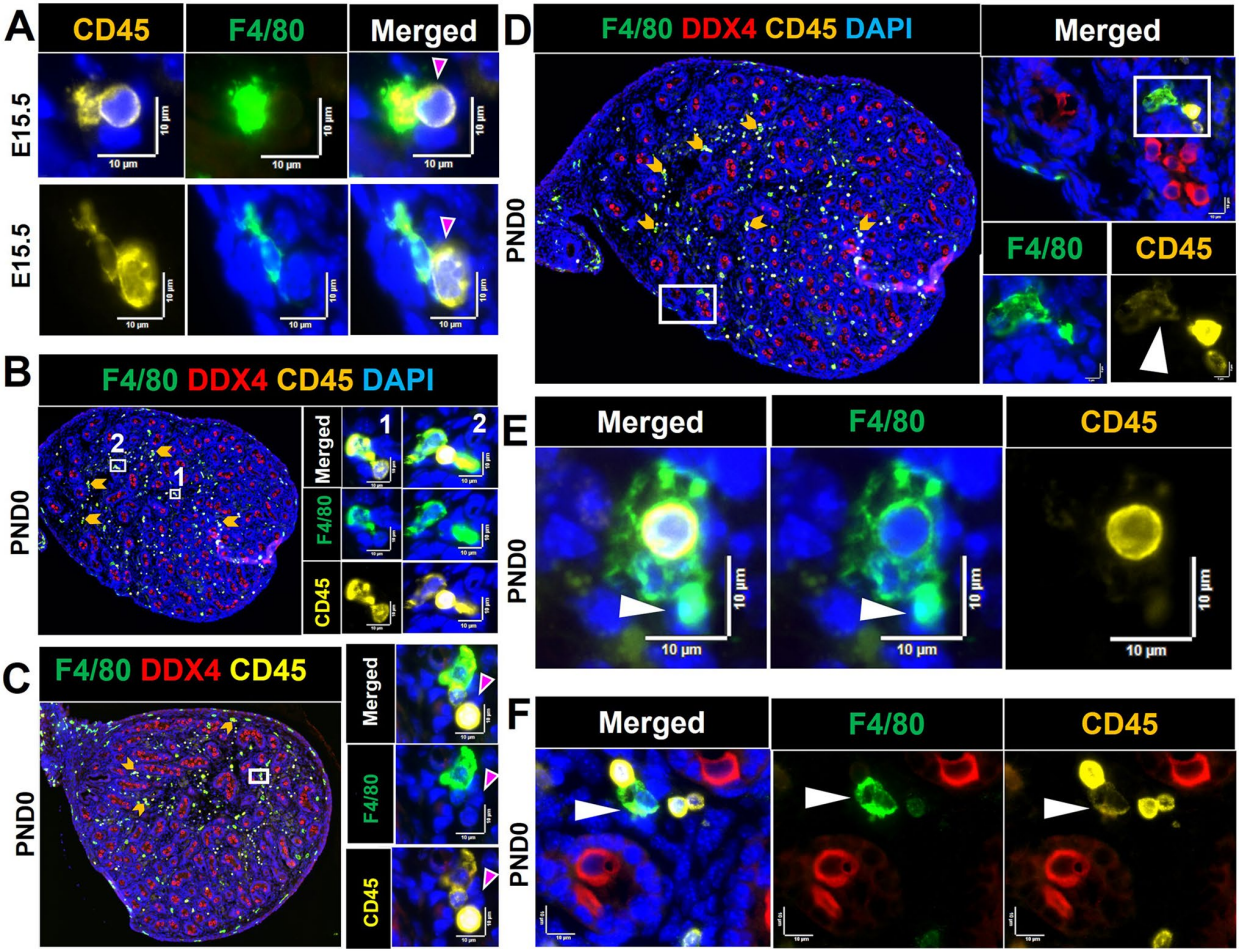
Contacts and co-localization of macrophages (CD45^+^/F4/80^+^) with immune (CD45^+^/F4/80^−^) and other cells (CD45^−^) in fetal and newborn mouse testes.** A** E15.5 testis. Contact between CD45^+^/F4/80^+^ and CD45^+^/F4/80^−^ cells. *Upper panels* CD45^+^/F4/80^−^ cell (*pink arrow*) contacting CD45^+^/F4/80^+^ cells. **B–F** PND0 testis. **B** (1) Co-localization of a CD45^+^/F4/80^+^ and CD45^+^/F4/80^−^ cell; (2) two CD45^+^/F4/80^+^ cells flanking a CD45^+^/F4/80^−^ cell. **C** A polymorphonuclear (PMN) CD45^+^ cell flanked by a CD45^+^/F4/80^+^ and a CD45^+^/F4/80^−^ cell within a cluster of CD45^−^ cells (e.g., *pink arrow*). **D** Co-localisation of a CD45^+^/F4/80^+^, a CD45^+^/F4/80^−^ and a PMN; *white arrow* indicates the dim level of CD45 on CD45^+^/F4/80^+^ cell. **E** A CD45^+^/F4/80^−^ cell engulfed by a CD45^+^/F4/80^+^ cell; *white arrow* indicates the very dim CD45^+^ signal on a CD45^+^/F4/80^+^. **F** Contact between a CD45^+^/F4/80^−^ cell with a CD45^Dim^F4/80^+^ cell (*white arrow*). *White boxes* in low-magnification images on the *left* in **B**–**D** correspond to high-magnification panels on the *right*. *Marker colors* are indicated above each panel set

The appearance of close contacts between non-macrophage cells, identified as CD45^+^/F4/80^−^ and CD45^−^/F4/80^−^ cells, and macrophages (CD45^+^/F4/80^+^) was a consistent observation, with 2–6 cells typically identified within an individual cluster, and between 2 and 6 clusters present in each section from the E15.5 and PND0 samples (Fig. 3). Polymorphonuclear cells (PMNs) were detected within some clusters (Fig. 3B, C) located in interstitial areas or around cords. The CD45 signal was dim on large and elongated F4/80^+^ cells (Fig. 3D–F). The low quantity mean levels during fetal testis development of *Ptprc*, encoding CD45, compared to *Adgre1*, encoding F4/80, may reflect the low level of CD45 on F4/80^Hi^ cells (Fig. 1A′).

**Fig. 4:**
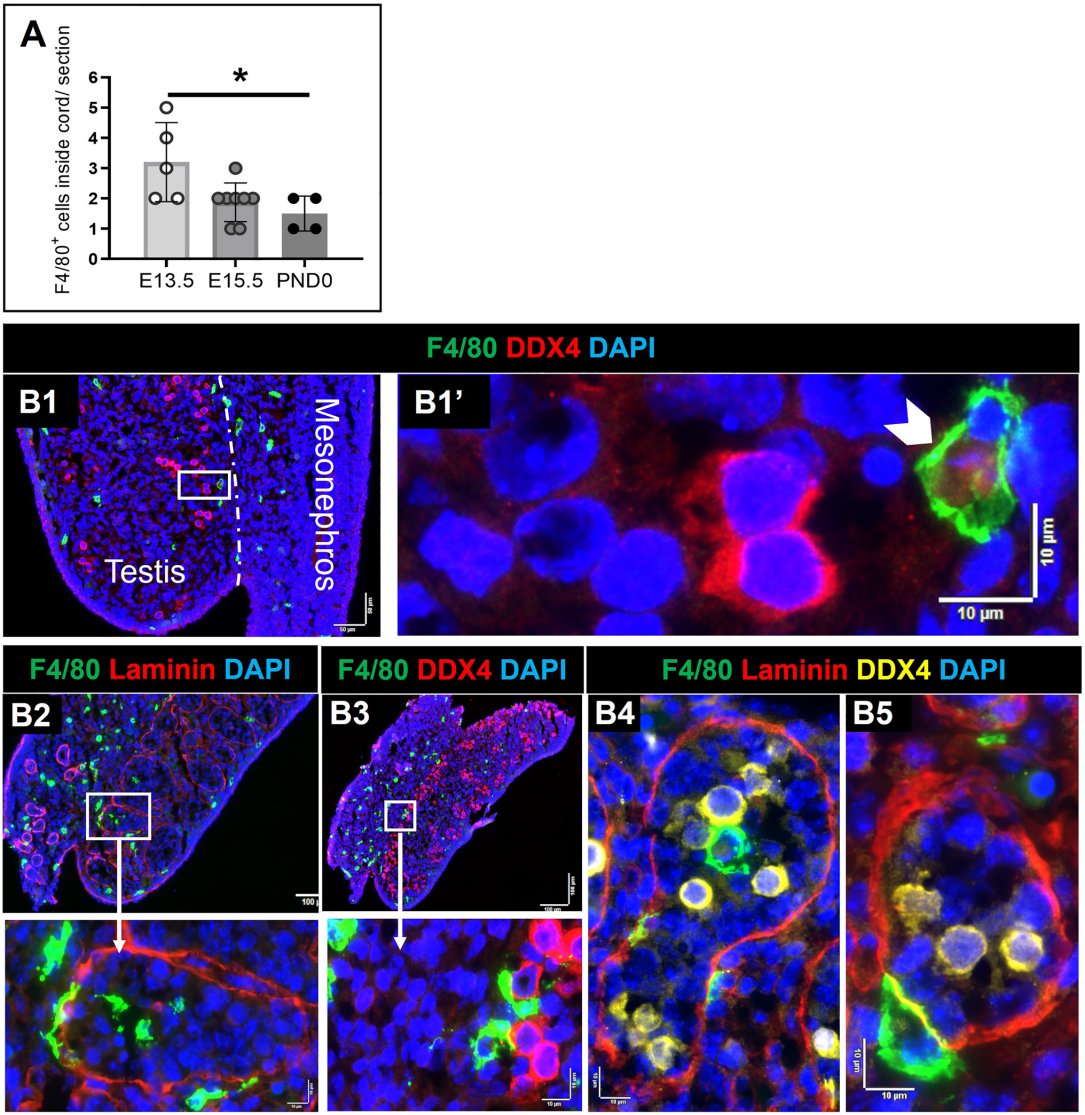
Proximity of macrophages (F4/80^+^) with germ cells (DDX4^+^) and cord basement membrane (laminin) in the E13.5 testis. **A** The number of F4/80^**+**^ cells inside cords per section, from E13.5 to PND0. Each *point* represents the average of 3 sections per biological sample. *Asterisk* indicates *p* ≤ 0.05. ** B1** Germ cell engulfed by a macrophage on the side of the testis adjacent to the mesonephros (*white arrow*). **B2**–**B4** Macrophages inside cords. **B5** Macrophage in close contact with cord basement membrane. *White boxes* in high-magnification panels refer to low-magnification images to the *right* or *below*). *Dotted white line* in **B1** denotes border between testis and mesonephros. *Marker colors* indicated above for each panel set

**Fig. 5:**
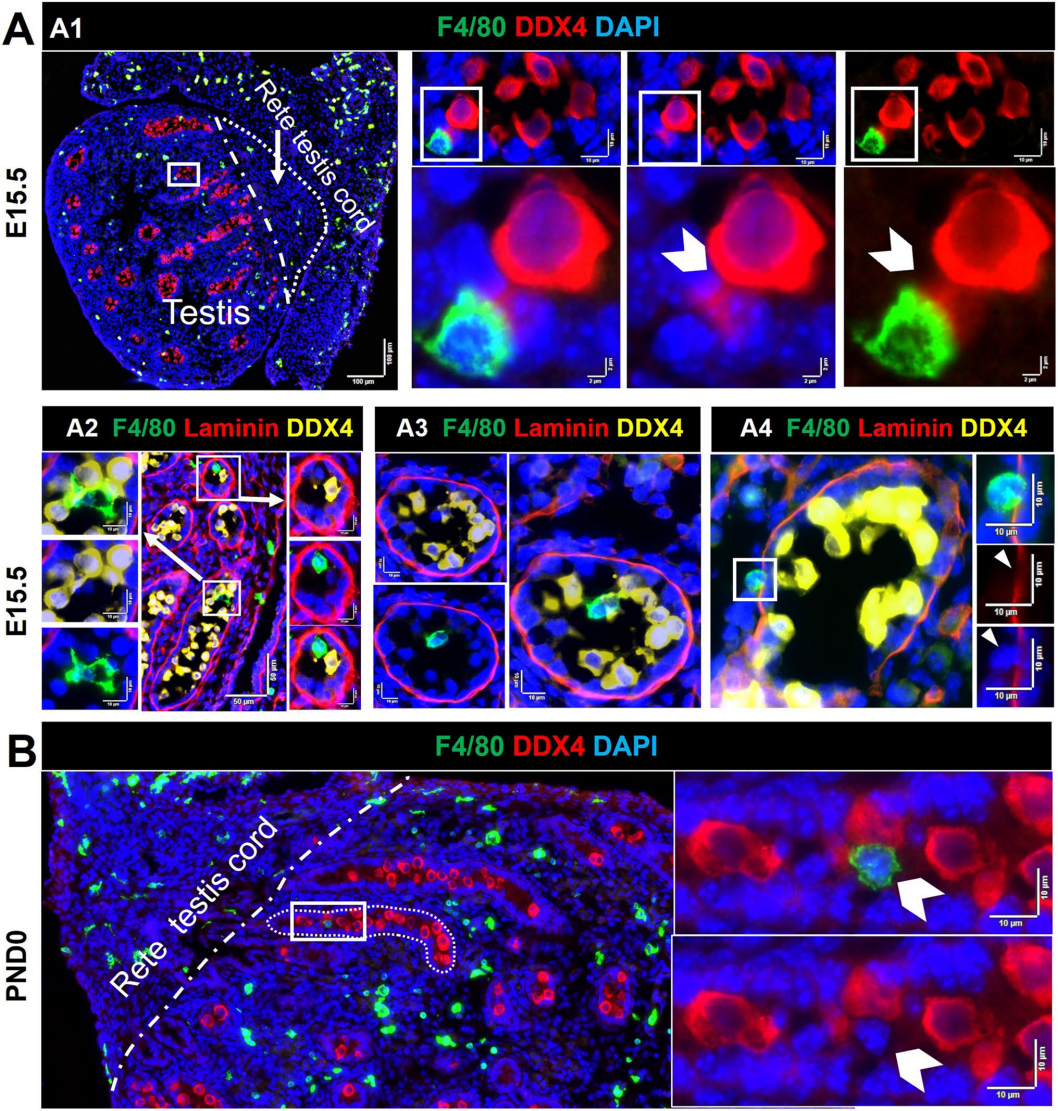
Proximity of macrophages (F4/80^+^) to germ cells (DDX4^+^) and cord basement membrane (laminin) in E15.5 and PND0 testes. **A1** Germ cell in close contact with a macrophage, on the side of the testis adjacent to the mesonephros (*white arrow*). **A2**–**A3** Macrophages inside cords. **A4** A macrophage positioned in the middle of the cord basement membrane (*white arrow*). **B** Co-localisation of a macrophage with germ cells in cord center (*white arrow*). *White boxes* in high-magnification panels refer to adjacent low-magnification images (**A1**–**A3**). *Dotted white line *in **A1** and **B** denotes testis–rete cord testis border. *Marker colors* are indicated above each panel set

The broad distribution of F4/80^+^ cells was variously documented inside the lamina propria, adjacent to cords, inside cords, and in close contact with germ cells (Figs. 4, 5). Macrophages were observed in contact with germ cells inside cords at E13.5 and E15.5 (and rarely at PND0), with between 1 and 5 in every section (Fig. 4A); this suggests that close contact of macrophages and germ cells inside cords may be a common phenomenon during this period of development (Supplementary Figure S5). At E13.5, apparent macrophage-engulfed germ cells were only rarely observed (between 0 and 1 per section), and these were typically close to the testis border with the mesonephros (Fig. 4B1). Macrophages inside cords were observed both close to the basement membrane and in the cord center at E13.5 (Fig. 5A2–4; Supplementary Figure S5; between and 2 and 5 per section). Similarly, at E15.5, macrophages were in contact with germ cells near the basement membrane of cords and inside cords (Fig. 5A; between and 1 and 3 per section). At PND0, there were few macrophages inside cords (Fig. 5B; between and 1 and 2 per section). Whether the F4/80^+^ cells contacting the basement membrane at E13.5 and E15.5 are in transit between the cords and interstitium, and whether they form a stable population around the testis cords, is unknown.

### CD206^+^ macrophages are predominant at E13.5, but not at PND0

**Fig. 6:**
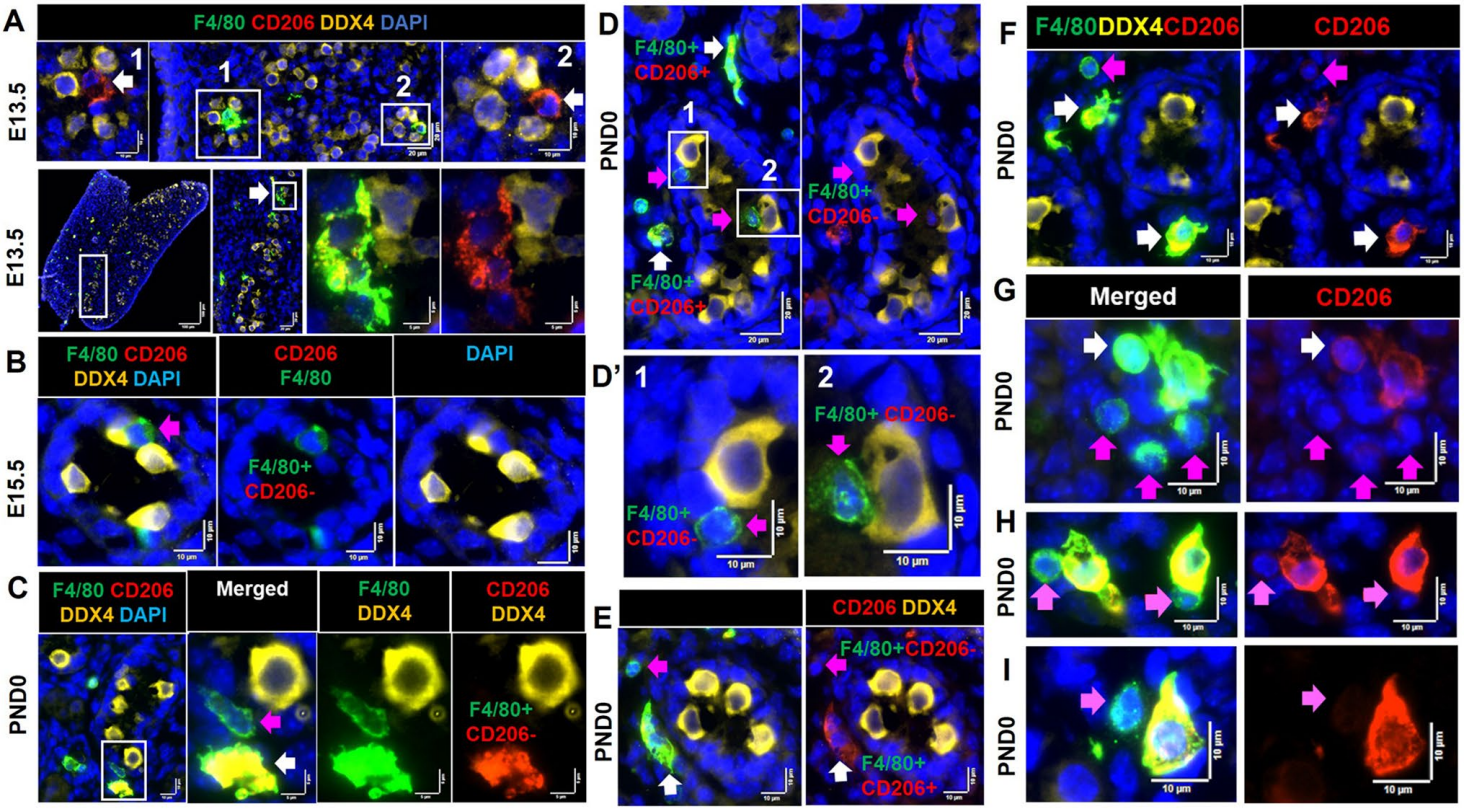
Features of F4/80^Hi^/CD206^+^ and F4/80^Int/Dim^/CD206^−^ cells in the E13.5 to PND0 mouse testis. **A** F4/80^Hi^ cells in close contact with germ cells inside cords were CD206^+^ at E13.5 (*white arrows*). **B** Small F4/80^Int^ cells in close contact with germ cells inside cords were CD206^−^ at E15.5 (*pink arrows*). **C** and **D** Small F4/80^Int^/CD206^−^ inside cords vs. large F4/80^Hi^/CD206^+^ cells outside cords at PND0. **E** and **F** A small F4/80^Int^/CD206^−^ cell in the interestituim (*pink arrows*) and two large F4/80^Hi^/CD206^+^ cell in the cord perimeter area (*white arrows*). **G** A cluster of F4/80^Hi^/CD206^+^ cells (*white arrow*) and small F4/80^Dim^CD206^−^ cells (*pink arrows*). **H** Contact between two large F4/80^Hi^/CD206^+^ cells with small, rounded F4/80^Int^/CD206^−^ cells (*pink arrows*). **I** Co-localisation a small F4/80^Int^/CD206^−^ and a large F4/80^Hi^/CD206^+^ cell. *Marker colors* are indicated above each panel set. *White boxes* on low-magnification images refer to the high-magnification panels

The predominance of CD206^+^ macrophages cells in the early fetal mouse testis up to E13.5 has been reported (DeFalco et al., 2014). To examine their relative frequency and position in later stages of fetal testis development, F4/80^+^/CD206^+^ and F4/80^+^/CD206^−^ cells were documented (Table 2). At E13.5, 81% of F4/80^+^ cells were CD206^+^, while, at PND0, this value had declined to half (49%). However, the absolute number of CD206^+^ macrophages per section increased during this interval. All F4/80^+^ cells inside cords at E13.5 were CD206^+^ (Fig. 6A), at E15.5, both F4/80^+^/CD206^−^ and F4/80^+^/CD206^+^ were seen (Fig. 6B), while at PND0, macrophages inside cords were CD206^−^ (Fig. 6C, D). Therefore, at PND0, F4/80^+^/CD206^+^ macrophages were detected across the tissue section, except for inside cords. The majority of macrophages located adjacent to cords were F4/80^+^/CD206^+^ (Fig. 6E, F). Interstitial macrophages appeared either as large and elongated F4/80^Hi^/CD206^+^ or small, rounded F4/80^Dim^/CD206^Dim/−^ subtypes (Fig. 6G).

**Table 2:** Quantification of CD206 positive and negative macrophage in E13.5 to PND0 testis sections

Age	E13.5 *n* = 5		E15.5 *n* = 8		PND0 *n* = 4	
Number of F4/80^+^ cells per cross section Mean ± SD (min-max)	19.6 ± 8.6 (7–31)		39.5 ± 11.4 (30–60)		141.5 ± 7.3 (136–152)	
Number of F4/80^+^CD206^+^ and F4/80^+^CD206^−^ cells per cross section Mean (min–max)	CD206^+^	CD206^−^	CD206^+^	CD206^−^	CD206^+^	CD206^−^
	16 (7–26)	3.6 (0–10)	24 (16–37)	15.43 (13–23)	70.5 (68–74)	70.5 (68–74)
Proportion of F4/80^+^ cells also expressing CD206^+^	81%		60%		49%	
Number of MHCII^+^/F4/80^−^ cells per cross section Mean ± SD (min–max)	0.2 (0–1)		4.1 ± 3 (0–10)		15 ± 4.9 (9.7–19.8)	

The values presented are the average of the number of cells counted in each of 4 cross-sections per sample, to emphasize the expanding number of these cells over this period of testis growth

*n* number of independent biological samples in each group

**Fig. 7:**
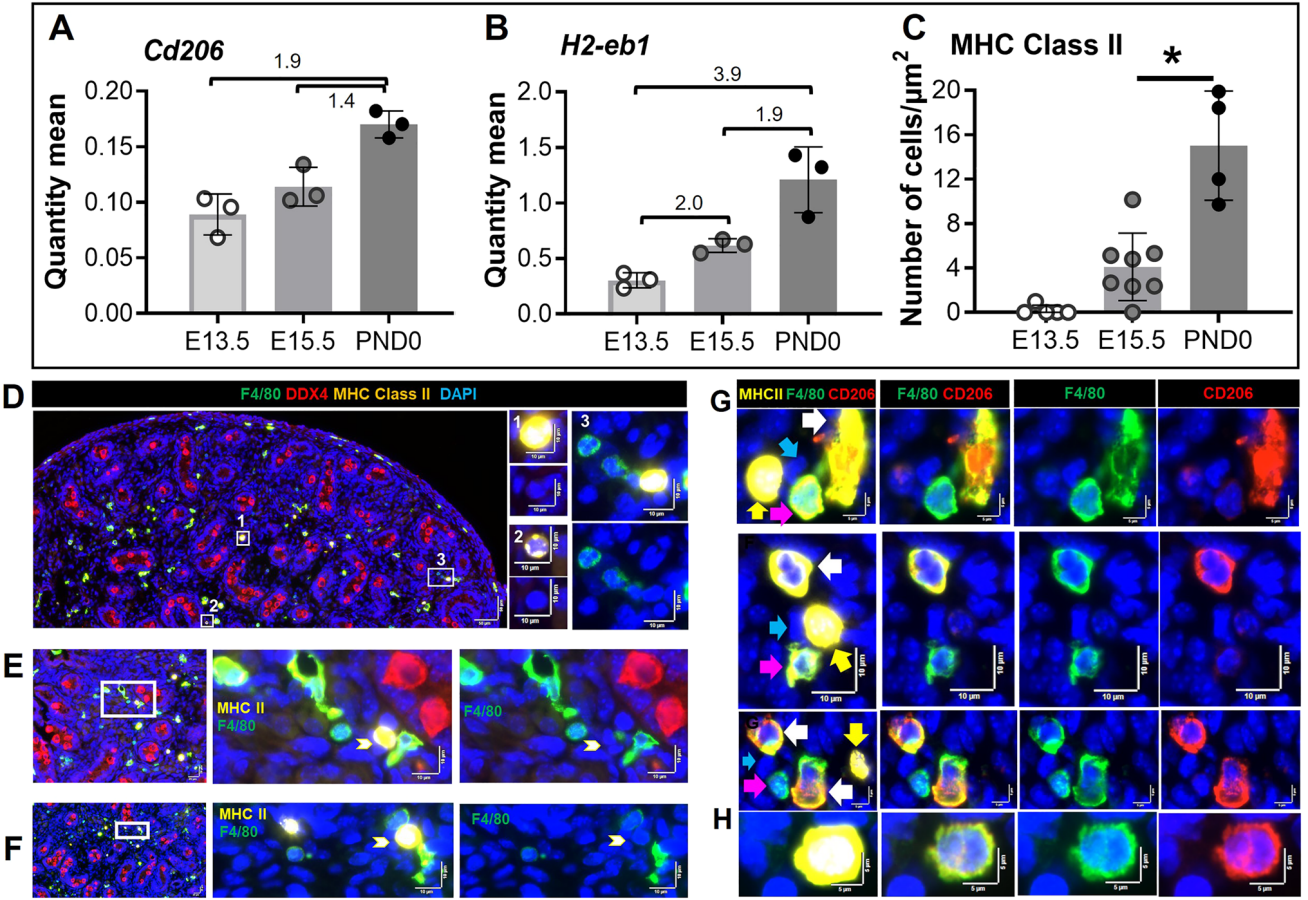
Features of MHC Class II^+^ cells in the newborn (PND0) mouse testis. **A** *Cd206* transcript levels. **B** *H2-eb1* (encoding MHC class II) transcript levels. RT-qPCR results reported as quantity mean of transcripts normalized to RPLP0; fold-change values between age groups indicated. **C** The density of F4/80^−^/MHCII^+^ cells at each age, from E13.5 to PND0 testis cross-sections. **D** Large (≥ 10 µm) (**1**) or small (≤ 10 µm) F4/80^−^/MHCII^+^ cells (**2**, **3**). **E, F** Contacts between small F4/80^−^/MHCII^+^ and F4/80^+^/MHCII^−^ cells. **G** Three examples of clusters of large F4/80^Hi^/CD206^+^ cells (*white arrows*), small F4/80^Int^/CD206^−^ cells (*pink arrows*) and small F4/80^−^/MHCII^+^ cells (*yellow arrows*). Additional, unidentified cells present in clusters are indicated (*blue arrows*). **H** A rare, single F4/80^+^/CD206^+^/MHCII^+^ cell. In **D**–**F** *White boxes* on low-magnification images refer to the high-magnification panels. *Marker colors* are indicated above each panel set

A variety of macrophage cell–cell contacts were frequently observed in the PND0 testis, including contact between two CD206 positive macrophages, one CD206 positive macrophage and one negative, and a cluster of CD206 positive and/or negative macrophages (Supplementary Figure S6A; Fig. 6G, H). Most CD206^+^ macrophages had an elongated, rather than rounded, shape (Supplementary Figure S6). The most common macrophage population in the perimeter area and in the mesonephros/epididymis of fetal and newborn testes was F4/80^+^/CD206^+^ (Supplementary Figures S6C, S7A, B). *Cd206* transcript levels elevated modestly (1.9-fold) from E13.5 to PND0 (Fig. 7A).

### Absence of MHC class II^+^ macrophages in fetal and newborn testis

The transcript encoding MHC class II *(H2-eb1)* increased 3.9-fold from E13.5 to PND0 (Fig. 7B). Counting of IF sections revealed that F4/80^+^/MHCII^+^ cells were absent to rare at E13.5, but detectable by E15.5 (0–3 per section), and significantly increased by PND0 (8–16 per section). *H2-eb1* transcript (encoding MHCII) levels roughly doubled from each 13.5 to E15.5 and from E15.5 to PND0 (1.9-fold) (Fig. 7B, C; Table 2). Based on size, the population of F4/80^−^/MHCII^+^ cells could be divided into less than or equal to 10 µm, or greater than 10 µm diameter (Fig. 7D; Supplementary, Figure S7C). Contact between F4/80^−^/MHCII^+^ cells with one or more F4/80^+^ cells was a common observation at PND0 (Fig. 7E, F). Also, we identified clusters of large F4/80^Hi^/CD206^+^, small F4/80^Dim^/CD206^−^, and F4/80^−^/MHCII^+^ cells (Fig. 7G). Almost all F4/80^+^ cells from E13.5 to PND0 were MHCII^−^; however, we detected extremely rare and infrequent (0–1 per section) F4/80^+^/CD206^+^/MHCII^+^ cells at PND0 (Fig. 7H).

### CD3^+^ cells populate the newborn testis

**Fig. 8:**
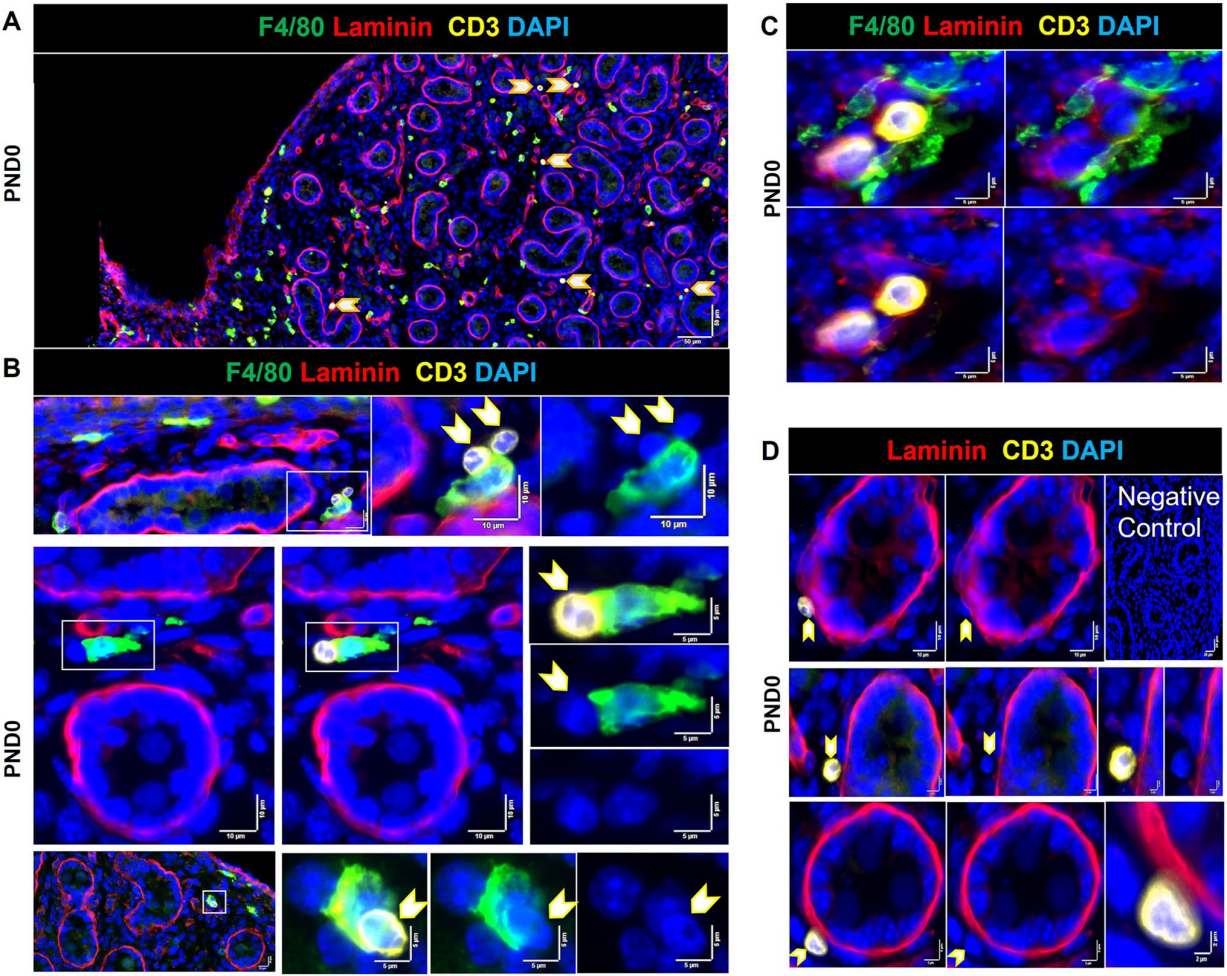
Location and cell–cell interactions of CD3^+^ cells (T cells) in the PND0 mouse testis. **A** Testicular T cells at PND0 indicated by *arrowheads*. **B** T cells appearing to be dividing. **C** Co-localisation of macrophage (F4/80^+^) and CD3^+^ cells (*arrowheads*). *White boxes* on low-magnification images refer to the high-magnification panels on the *right*. **D** A macrophage surrounding two CD3^+^ cells in close contact with the basement membrane (laminin^+^). *Marker colors* are indicated above each panel set.

Because almost half the population of CD45^+^ cells in the PND0 testis were not F4/80^+^, we reasoned that immune cell types other than macrophages must populate the fetal and newborn testis. As the rare detection of a CD3^+^ cell in the E12.5 mouse testis was previously reported (DeFalco et al. 2014), we addressed the possibility that T cells may be contributing to the changing immune cell density. By immunofluorescence, the commonly used pan-T cell marker, CD3^+^, and its encoding transcript were not detected at E13.5, although a few cells were detectable (0–1 cell/section) at E15.5 (Supplementary, Figure S3). In the newborn testis, however, CD3^+^ cells were present in the perimeter region, interstitium, and positioned adjacent to cords (Fig. 8A). Contact between one or two CD3^+^ cells and F4/80^+^, appearing as a cluster, was a frequent observation at PND0 (2–4 clusters/ section) (Fig. 8B, C). CD3^+^ cells were occasionally found adjacent to the cord membrane (Fig. 8D).

### Ly6G^+^ cell density increases dramatically through fetal testis development

**Fig. 9:**
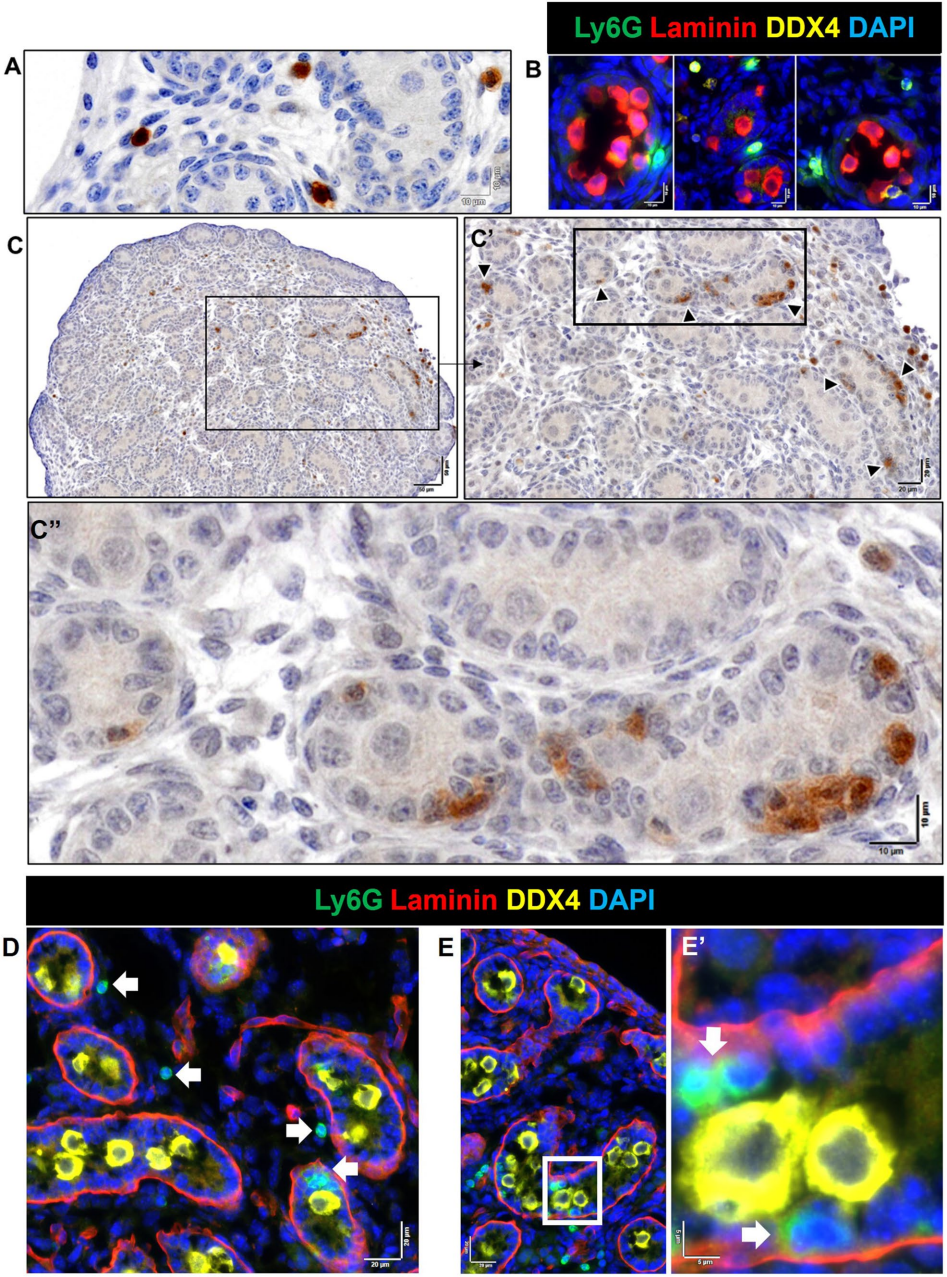
Localization and cell contacts of Ly6G^+^ cells (neutrophils) in fetal and newborn mouse testes. **A** Ly6G^+^ cells in the interstitium at PND0. **B** Ly6G^+^ cells at cord perimeter areas at E15.5. **C** Ly6G^+^ cells inside cords at PND0 (*dark arrowheads*). **D** Several Ly6G^+^ cells in close proximity are present in the interstitium, near the cord perimeter and inside cords (*white arrows*) at PND0. **E** Co-localisation of Ly6G^+^ cells and DDX4^+^ cells inside cords (*white arrows*) at PND0.* Marker colors* are indicated above each panel set. *Black and white boxes* on low-magnification images refer to the high-magnification panels

**Fig. 10:**
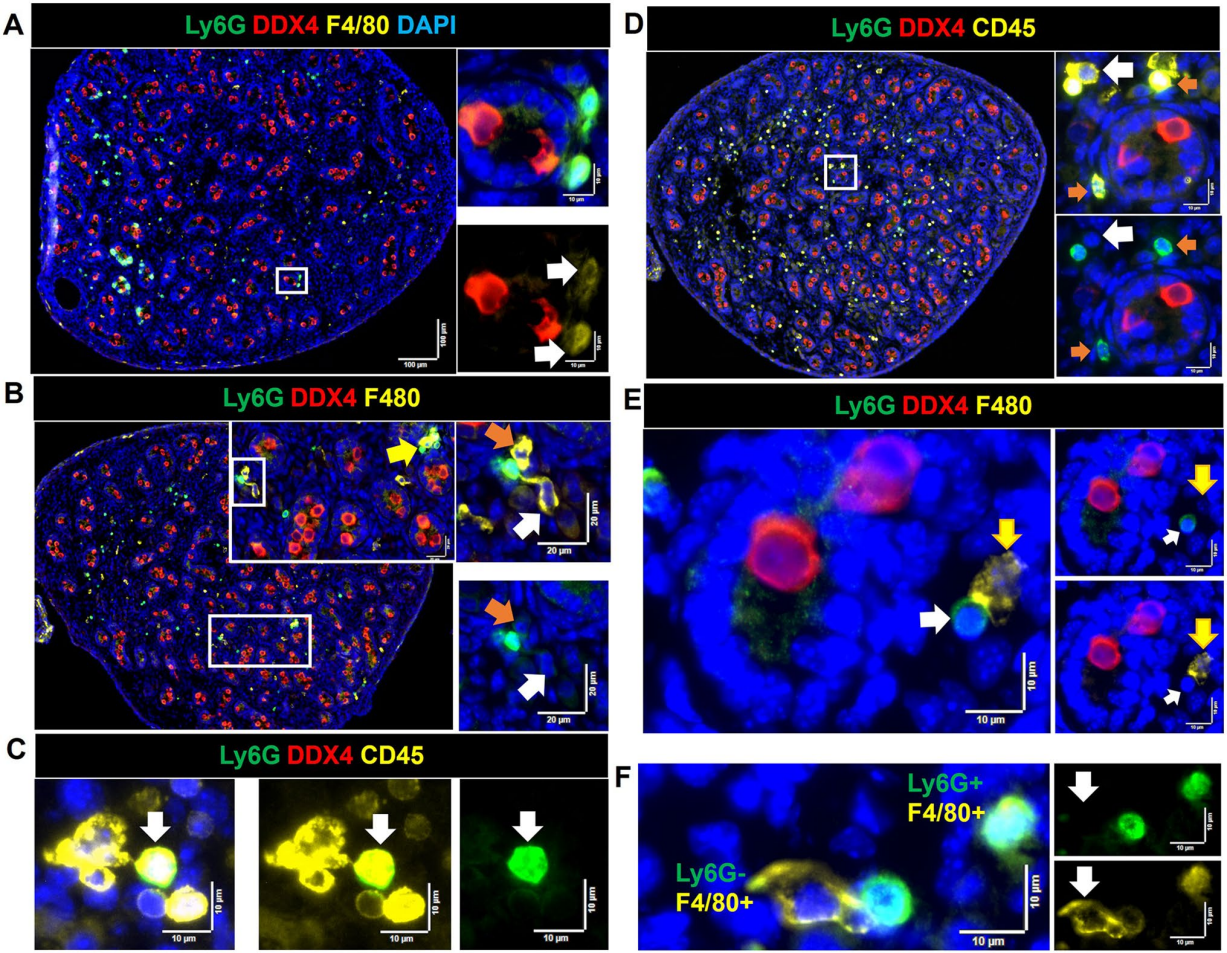
Interactions of Ly6G^+^ cells (neutrophils) with other cells in the newborn mouse testis (PND0). **A** Two Ly6G^+^ cells in the cord perimeter area displaying a dim F4/80 signal (*white arrows*). **B** A neutrophil in contact with two macrophages: one semi-rounded macrophage in the cord perimeter area (orange arrows) and another in the interstitial area (*white arrows*), and a grouping of Ly6G^+^ and F4/80^+^Ly6G^−^ cells (*yellow arrow*). **C** Co-localisation of a neutrophil (*white arrow*) with several CD45^+^ cells. **D** Co-localisation of two Ly6G^+^ cells with CD45^+^ cells at cord perimeter (orange arrows). Co-localisation of a CD45^+^/Ly6G^+^ and a CD45^+^/Ly6G^−^ cell in the interstitium (*white arrows*). **E** A Ly6G^+^ (*white arrow*) and a F4/80^+^Ly6G^−^ (macrophage; *yellow arrow*) attached to each other. **F** Two Ly6G^+^ cells displaying different F4/80 levels. *White boxes* on lower-magnification images refer to the high-magnification panels on the *right*. *Marker colors* are indicated above each panel set or directly on figures

**Fig. 11:**
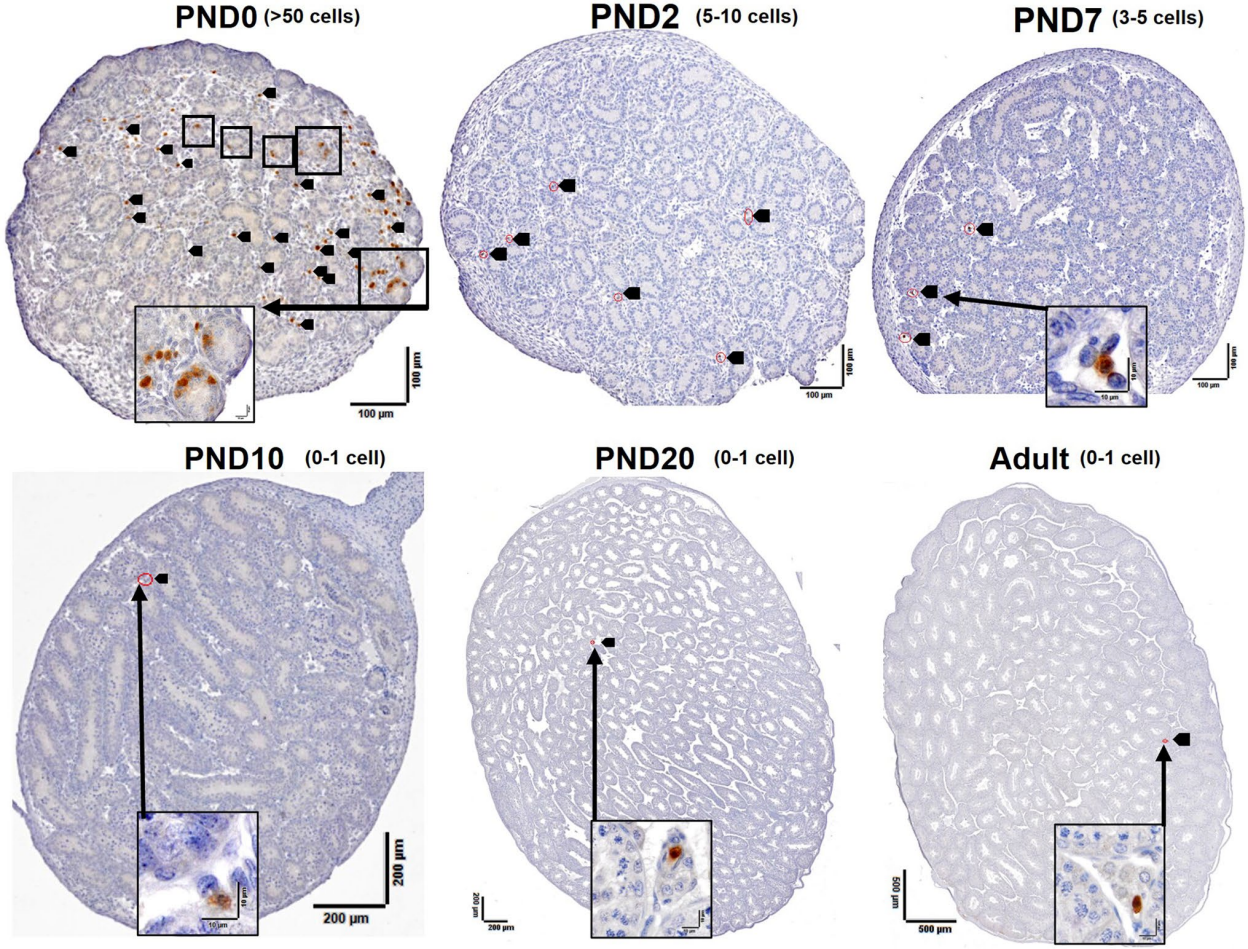
Ly6G^+^ cells (neutrophils) in mouse testis sections from birth to adulthood. Higher-magnification images in *black boxes* show Ly6G^+^ cells inside cords at PND0. *Red circles with black arrows* indicate all neutrophils detected in each section, except for the PND0 sample which had more than 50 labeled cells. The minimum and maximum number of Ly6G^+^ cells in the central section of each of 3 testes per age testis shown in parentheses. After day 10, Ly6G^+^ cells were rare to absent

At the first stage of this investigation, we checked for the presence of neutrophils using anti-MRP14/S100A9, which marks both neutrophils and monocytes (Wang et al. 2021), and observed positive cells with high frequency in the newborn testis. We then examined the potential presence of neutrophils, identified here as Ly6G^+^ cells, as this cell type is commonly linked to tissue remodeling. The highly-specific Ly6G marker recognizes all mouse neutrophil subtypes, but has also been reported on some macrophages (Wang et al. 2021). Their frequency was very low at E13.5 (0–3 Ly6G^+^ cells per section) and had increased substantially by E15.5 (27–67 per section) (Fig. 1A, A′). The Ly6G^+^ population increased even further, to represent more than half of the CD45^+^ cells, at PND0 (80–100 per section; Fig. 1C). Ly6G^+^ cells were detected in the interstitium, and were both next to and inside cords, but they were rare to absent in the perimeter region. The distribution of the increasing number of these cells at E15.5 and PND0 appeared asymmetric across the section: they were predominantly detected in the section interior and were rarely in the perimeter (Supplementary Figure S8). This contrasts with the macrophage distribution, which shifts from the perimeter to the interior between E13.5 and PND0 (Table 1). Ly6G^+^ cells were frequently noted inside some cords adjacent to germ cells only at PND0 (Fig. 9). Ly6G^+^ cell co-localization with macrophages (F4/80^+^) and/or with other immune cells (CD45^+^ cells lacking either F4/80 or Ly6G), including as clusters adjacent to cords and/or in interstitial areas was also regularly observed (Fig. 10). Some Ly6G^+^ cells in the fetal testis showed a dim F4/80 signal. This was documented using two independent antibodies, one which was unconjugated (in-house) and the other directly conjugated (commercial), leading to our conclusion that two distinct Ly6G^+^ cell populations are present, one lacking F4/80 and one with a low level of this protein (Fig. 10F). The presence of more than 50 Ly6G + cells per section in the newborn testis coincided with the robust elevation of *Lyg6* between E15.5 and PND0, signifying a clear answer to the question of what cell type became prevalent alongside macrophages at this age*.* This increase around the time of birth was followed by a significant drop in their number afterwards, with 5–10 cells per section identified at day 2, 3–5 cells at day 7, and 0–1 from day 10 to adulthood (Fig. 11).

## Discussion

Although the importance of macrophages for the initial stages of testis cord formation in the fetal testis is widely accepted, there is a gap in understanding which other immune cells contribute to testis growth after the cords first form, up to shortly after birth when spermatogenesis is initiated. In addition, macrophage origins and roles in postnatal testis growth and ongoing adult spermatogenesis in homeostatic conditions remain key discussion points in the field (DeFalco et al. 2014; Mossadegh-Keller et al. 2017; Mossadegh-Keller and Sieweke, 2018; Lokka et al. 2020; Wang et al. 2021). The present study extends outcomes from previous investigations of fetal testis macrophages by examining their phenotype and distribution in the interval following sex determination through to birth in the mouse testis. The documentation of CD3^+^ and Ly6G^+^ cell emergence is an important finding which highlights the dynamic nature of immune cell subpopulations in the fetal testis, revealing their close association with germ cells. The potential presence of B cells in the fetal mouse testis at E13.5 was examined previously using the B220 marker in sections, and no B cells were detected (DeFalco et al. 2014). We also checked for B cells using CD19 as a marker by IF, and no positive cells were identified at any age (data not shown). An important caveat to this study is the well-documented heterogeneity of each immune cell population, including macrophages, granulocytes, and T cells, so that the detection of a single marker must be interpreted with caution. For example, T cell lineage markers have been reported on macrophages and other myeloid cells (Chávez-Galán et al. 2015), and Ly6G is found on some macrophages (Wang et al. 2021) in experiments using flow cytometry. However, histological evaluation of the cells expressing each of these markers remains an effective approach to providing cell type identification.

### The changing landscape of immune cell subpopulations in the fetal mouse testis

Quantitative analysis of sections examined using indirect immunofluorescence documented the increasing density of immune cells (CD45^+^), highlighting changes in the relative contributions of macrophages (F4/80^+^), T cells (CD3^+^), and neutrophils (Ly6G^+^) (Fig. 1). While each subpopulation increases significantly in density between E13.5 and PND0, indicating their numerical elevation, the proportion of F4/80^+^:CD45^+^ cells decreased significantly, while Ly6G^+^ cells were rare to absent at E13.5. Thus, while our E13.5 data agree with the earlier report that 90% of CD45^+^ cells in the E12.5 testis are macrophages (DeFalco et al. 2014), it was surprising that, by PND0, about half of CD45^+^ cells in the testis were Ly6G^+^. The detection of both Ly6G^+^ and CD3^+^ cells in the newborn testis indicates that the immune milieu is altered dramatically by the time shortly after birth that spermatogenesis commences.

### Two phenotypes of fetal testicular macrophages

Based on a previous ontological investigation, F4/80^Hi^ macrophages detectable before E14.5 are known to be yolk sac-derived, while F4/80^Int^ are fetal liver-derived and emerge by E16.5 (Lokka et al. 2020). In agreement with this, we detected only elongated F4/80^Hi^ macrophages at E13.5, while, at E15.5 and PND0, both elongated F4/80^Hi^ and small, rounded F4/80^Dim^ macrophages were noted. The dim expression of CD45 on testicular macrophages at E13.5 is consistent with previous reports (DeFalco et al. 2014; Lokka et al. 2020), while higher levels were observed at E15.5 and PND0. Adult kidney and microglia also contain two CD45^+^ macrophage subpopulations, with “intermediate” and “high” signals, which are proposed to have different origins (Lee et al. 2018; Rangaraju et al. 2018). The low level of CD45 on testicular macrophages during fetal life indicates that this is not an appropriate marker for first gating in flow cytometry or cell sorting techniques to study testicular macrophages at these developmental stages. In contrast, CD45 on Ly6G^+^ cells which appeared to be neutrophils was present at high and readily detectable levels.

The previous report of fetal testicular macrophages concentrated near the gonad–mesonephros boundary at E10.5 and E13.5 demonstrated that a substantial proportion of macrophages originate and enter the testis from the mesonephros (DeFalco et al. 2014). We also observed macrophages at E13.5 and E15.5 on the mesonephros side of the testis, and note that they frequently appeared to be attached to and crossing through the capsule. Intriguingly, these later profiles appeared highly similar to leukocyte extravasation (Muller, 2013). The subsequent redistribution of macrophages from perimeter to the interior region of the testis contrasts with the localization of neutrophils. Ly6G^+^ cells are rare at E13.5 but increased from E15.5 onwards; they appeared asymmetrically distributed across the fetal testis sections at E15.5. Also, the lack of macrophages detected inside the lumen of blood vessels at E13.5, E15.5, and PND0 contrasts with the frequent observation of Ly6G^+^ cells inside vessels, particularly at PND0, a distinction that merits further careful analysis.

While confirming the previous report of F4/80^+^ polymorphonuclear cells in the fetal and newborn testis (Lokka et al. 2020), we also identified some Ly6G^+^ granulocytic cells, most likely neutrophils, that were F4/80^+^. In agreement with previous investigations, no MHC class II^+^ macrophages were detected in the fetal testis, with only rare CD206^+^/MHCII^+^ macrophages (0–1 per section) at PND0 (Mossadegh-Keller et al., 2017; Lokka et al. 2020; Wang et al. 2021). The main population of E13.5 macrophages, consisting of large, elongated, CD45^Dim^/F4/80^Hi^/CD206^+^/MHCII^−^ cells, became less prominent with the emergence of smaller, rounded CD45^Int^/F4/80^Dim^/CD206^−^/MHCII^−^ cells at E15.5. This may reflect the imperative for a shift from a predominantly immunosuppressive macrophage population towards one that includes more immune-reactive macrophages at birth. These findings consolidate the understanding that there are at least two distinct populations of macrophages in newborn mouse testis, as well as several other immune cell types.

### Cell–cell interactions of immune cells in the fetal testis

The full functional significance of the close localization and apparent contact between different immune cell subsets is unknown, but may include roles in fetal testis morphogenesis, immune regulation, and germ cell removal and development, all of which are integral to the foundations of male fertility. The phagocytosis of germ and Sertoli cells by macrophages in E11.5 and E12.5 mouse testes has been previously demonstrated (DeFalco et al. 2014), and we also observed germ cells outside the cords apparently engulfed by macrophages at the mesonephro–testis border at E13.5. Also consistent with the report of macrophages inside testis cords at E11.5 and E12.5 (DeFalco et al. 2014), and in accord with the detection of macrophages inside cords up to 2 weeks after birth (Lokka et al. 2020), we found macrophages inside cords, but at a declining incidence from E13.5 to birth. In contrast, Ly6G + cells were observed inside cords, but only at PND0. We also observed immune cells in close contact with other immune cell types from E13.5 to PND0, with between 4 and 10 immune cell clusters in each section, appearing more frequently with advanced age development. While the functional significance of these contacts is unknown, we speculate that macrophages may process germ cell antigens that are subsequently exposed to regulatory T cells for induction and maintenance of immune tolerance during adult life (Tung et al. 2017; Fijak et al. 2018; O'Donnell et al. 2021), or they may produce factors, such as activins or CSF-1, that influence germ and/or Sertoli cell development, as identified for the adult mouse testis (DeFalco et al. 2015). Based on others’ observations from early fetal testis development (DeFalco et al. 2014; Mossadegh-Keller et al 2017), and long-standing evidence of close structural and functional relationships between macrophages and Leydig cells in postnatal testes (Hutson 1992; Gaytan et al. 1994; Fadel and Sarzotti 2000), we acknowledge that the important interactions between these immune cells and the developing Leydig cells and vasculature may be highly relevant to identifying points of vulnerability that affect postnatal testis growth and function.

### Testicular CD3^+^ cells

We observed few CD3^+^ cells at E15.5, aligned with the report of DeFalco et al. (2014). This was unsurprising, because T cell development occurs late in fetal life, while the first thymic T cells are detectable in murine lymph nodes at E18–E20 (Fadel and Sarzotti 2000). Several T cells in the PND0 testis appeared to contact macrophages. However, the interaction of macrophages and T cells is not likely to be through MHC class II (*H2-Eb1*), because almost all macrophages at PND0 appeared to lack this marker. Most of the small number of MHCII^+^ cells in PND0 testes were F4/80^−^, indicating either that another antigen presenting molecule is present on testicular macrophages, or that antigen presentation through MHCII is not the function of T cell–macrophage interactions at PND0. The finding of T cells in the fetal testis, both adjacent to cords and in interstitial regions at PND0, suggests that, similar to adult macrophages (DeFalco et al. 2015; Mossadegh-Keller et al. 2017), there are at least two functionally distinct subpopulations.

### Ly6G^+^ cells in the fetal testis

Neutrophils constitute a key Ly6G^+^ cell population that plays critical roles in antibacterial immune responses, inducing inflammation, regulation of the hematopoietic niche, and angiogenesis, but their role in morphogenesis during development is much less well defined (McGrath et al. 2015; Christoffersson and Phillipson et al. 2018; Casanova-Acebes et al. 2018; Carnevale et al., 2020). An early report indicated that neutrophils are not detected in the adult rodent testis under normal condition, but can enter from the blood supply in pathophysiological conditions (O‘Bryan et al. 2000). Neutrophils have reported in mouse and rat testes with infection or testicular torsion, including in ischemia–reperfusion models (Lysiak et al. 2001, 2004; Celebi and Paul, 2008; Michel et al. 2016; Fijak et al. 2018), and in human testicular tumors (Akhtar et al. 1996), all relating to the adult testis.

In this report, we have made the remarkable identification of Ly6G^+^ cells as the second-most frequent immune cell population in the newborn mouse testis. The only previous evaluation of neutrophils in the fetal mouse testis, conducted using flow cytometry, identified 9% of CD45^+^ cells as neutrophils (Gr-1 + cells) at E12.5 (DeFalco et al. 2014), while our analysis of tissue sections identified neutrophils as rare to absent at E13.5 (0–3 cells per section). The difference between these two observations may reflect the sensitivity of these distinct approaches. Murine fetal neutrophils are present in both the liver and bloodstream by E11.5, and expand rapidly in both compartments; neutrophil phenotypes in the E12.5 liver are consistent with early through late neutrophil maturation stage phenotypes. The initiation of hematopoiesis in the bone marrow at E18.5 (Sugiyama et al. 2011) fits with the present finding that the Ly6G^+^ population increases in the mouse testis to compromize more than half of the CD45^+^ population by birth. The remarkable drop in Ly6G^+^ cell prevalence in the testis shortly after birth, and their near absence from the adult mouse testis, provide a strong indication that these cells serve a highly specific role in a particular stage of testis development. The abundance of Ly6G^+^ cells in this period of late fetal testis development, and at the onset of spermatogenesis, coincides with the movement of germ cells from the cord center to the perimeter, as they transform from gonocytes into spermatogonia. Their function may be related to a transient modification of the peri-cord environment or perhaps even the Sertoli cells, aligned with their known role in tissue remodelling (Peiseler and Kubes 2019).

In contrast to the finding that macrophages are frequent near the mesonephros at E13.5 and E15.5, Ly6G^+^ cells are mostly absent from this location. Their predominant location in the section interior, close to and inside vessels from E15.5 onwards, may reflect a role in vascularization of the fetal testis, essential for normal testis development, including formation of fetal Leydig cells, smooth muscle cells, and pericytes (Lysiak et al. 2001; Coveney et al. 2008; Kumar and DeFalco 2018). It will be important to consider their mechanism of recruitment to the developing testis, particularly as signaling molecules such as CXCL12, which recruits and maintains CXCR4^+^ neutrophils in lung (Carnevale et al. 2020), are also central to early germline development (Heckmann et al. 2018). A signaling link, such as the common potential for germ cells and neutrophils to respond to the Sertoli cell-derived products including CXCL12 and activin A, may explain why we observed neutrophils and germ cells co-located within the fetal testis.

Neutrophils have been increasingly identified as being heterogeneous in both structure and function, with a capacity for functional plasticity, and are predominantly reported in relationship to adult pathologies (Christoffersson and Phillipson, 2018; Carnevale et al. 2020). Changes in neutrophil maturation status are distinguished by their cell and nuclear shape (Hong, 2017). Here, we make the intriguing observation of Ly6G^+^ cells with two distinct phenotypes: those with round, kidney-shaped and segmented nuclei were detected at E15.5, while, at PND0, the dominant population was that with segmented nuclei. Based on their phenotypes, the Ly6G^+^ cells appeared to represent early- and mid-developmental neutrophil stages at E15.5 and the late differentiated stage at PND0. The application of additional markers will be required to track the developmental and functional trajectory of these cells within the nascent testis.

We observed further evidence of Ly6G^+^ cell heterogeneity in the early testis, indicating there are at least two populations: one clearly F4/80^+^ positive and one negative/dim for F4/80. While the transcript encoding F4/80, *Adgre1*, is detectable in murine neutrophils, the protein is not normally expressed on the cell surface (Sasmono et al. 2007). However, purified Ly6G^+^ cells express CSF-1R after overnight culture, and can subsequently differentiate to form F4/80^+^ macrophages in response to CSF-1 (Sasmono et al. 2007). It will be exciting to learn how the complex milieu of the developing testis may attract and instruct granulocyte maturation and function.

**Fig. 12:**
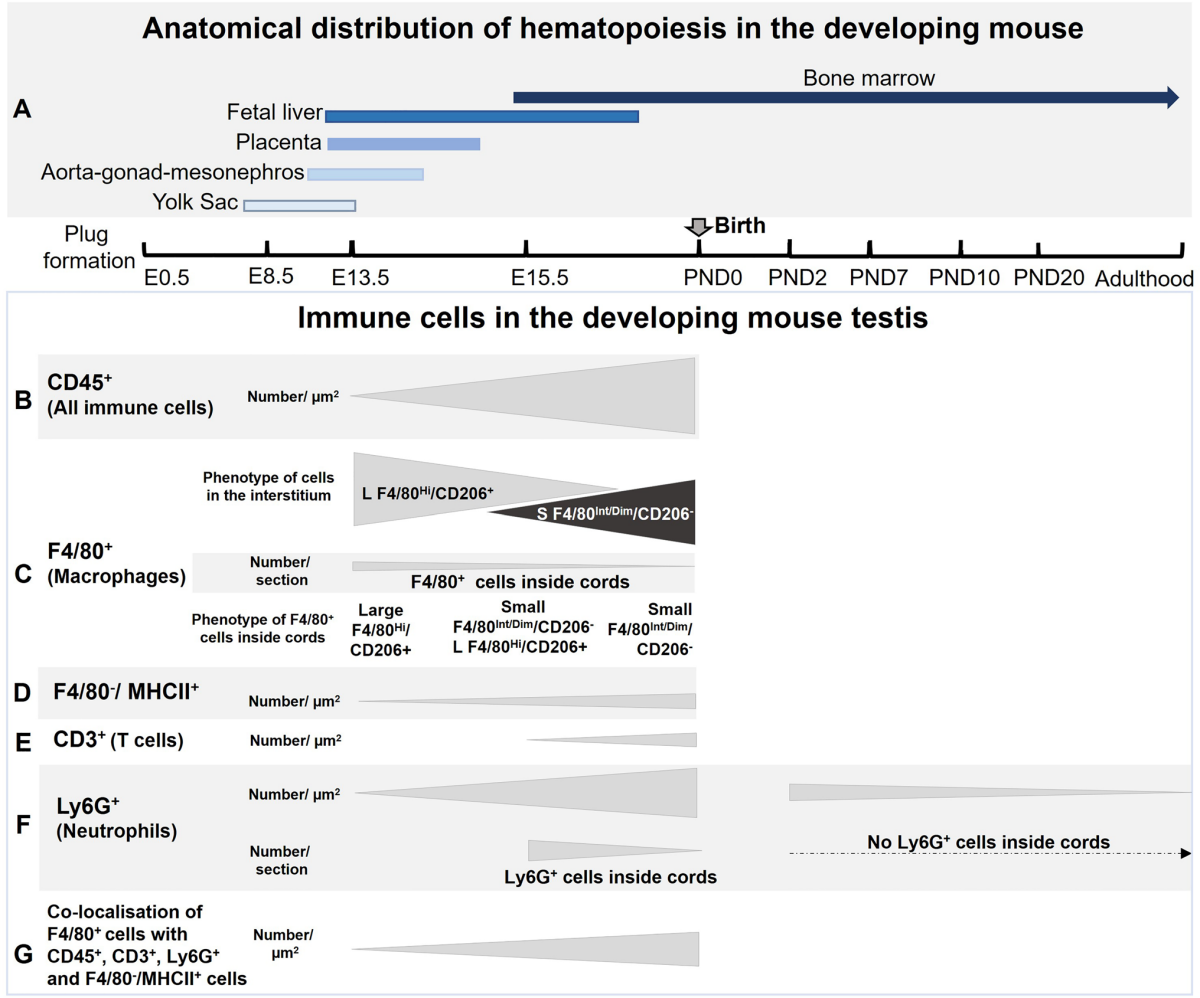
Anatomical distribution of hematopoiesis in the developing mouse and summary of study outcomes.** A** Primitive hematopoiesis starts at E7.5 in the yolk sac. At approximately E9.5, the aorta-gonad-mesonephros region accommodates hematopoiesis, followed by hematopoiesis in placenta and fetal liver at E11.5. The bone marrow is the predominant hematopoietic tissue from about E14.5 and throughout adulthood (Mikkola and Orkin, 2006; Hoeffel and Ginhoux, 2015; Mevel et al., 2019). **B** CD45^+^ cell density increased from E13.5 to PND0. **C** The phenotype of F4/80^+^ cells in the E13.5 testis interstitium was large and F/80^Hi^/CD206^+^, while, from E15.5, a small F/80^Int/Dim^/CD206^−^ population was detected. F4/80^+^ cells inside cords at E13.5 were exclusively large and F/80^Hi^/CD206^+^. Macrophages inside cords were large F/80^Hi^/CD206^+^ and small F/80^Int/Dim^/CD206^−^ at E15.5, and small F/80^Int/Dim^/CD206^−^ at PND0. **D** F/80^−^/MHC class II^+^ cells increased from rare/single at E13.5 to PND0. **E** CD3^+^ cells were not detected at E13.5. Single CD3^+^ cells were observed at E15.5, with scattered CD3^+^cells present at PND0. **F** Ly6G^+^ cells were rare at E13.5, while, by PND0, they represented half of the testicular immune cell population. A lower number of Ly6G^+^ were observed at PND7, but they were rare to absent after PND10 and in the adult testis. **G** F4/80^+^ cells in clusters with CD45^+^, CD3^+^, Ly6G^+^, F4/80^−^/MHCII^+^ cells were noted with increasing frequency from E13.5 to PND0

In conclusion, this study documents for the first time the progressive emergence of neutrophils and T cells, the frequency and localisation of macrophages, T cells, neutrophils, and their association with germ cells in the developing fetal mouse testis. The findings from this study are summarized in alignment with the timing of hematopoiesis in the murine yolk sac, fetal liver, and bone marrow (Mikkola and Orkin 2006; Hoeffel and Ginhoux 2015; Mevel et al. 2019) (Fig. 12). In particular, it establishes an understanding of the changing proportions of germline and immune cells that occur during fetal testis development in the mouse. Our understanding of the nature of the interactions between germline and immune cells is in its infancy. The production of Csf1 by macrophages as a modulator of spermatogonial fate (DeFalco et al 2014) and IL-6 by seminoma cells (testicular germ cell tumors; Klein et al. 2016) are two examples of the potential cross-talk mediators between testicular immune and germ cells in different physiological states. The capacity to identify potential reciprocal signaling pathways by inspecting data from single-cell RNA sequencing analyses offers a new strategy to generate hypotheses about the functional significance of the co-location of these cells in the highly dynamic fetal testis. By revealing the frequent, close cellular contacts between macrophages, T cells, and neutrophils during testis development, we provide information that will be key to understanding how immune cells function during development of the fetal mouse testis and influence maturation of the earliest male germ cells. Information gained from this study should be considered in the development of strategies to support in vitro germline growth, for example, by adding immune cells into scaffolds or organoids.

## Supplementary Information

Below is the link to the electronic supplementary material.

Supplementary file1 Supplementary Figure S1. Histology and approach to analysis of cell populations in sections. A. Cross sectional area (mm2) of mouse testis sections at E13.5, E15.5 and PND0. Each data point represents the average cross-sectional area of 4 sections from the testis of an individual animal, shown with mean and SD. B and C. Delineation of perimeter and internal areas of fetal mouse testis cross sections. Two different locations of F4/80+ cells are shown using (B) immunohistochemistry (brown stain) in PND0 testis and (C) immunofluorescence in E15.5 testis (macrophages (green), germ cells (yellow) and cord boundary (red)). Dotted lines denote the division between the section ‘perimeter’, between the testis capsule and edge of the outermost cord, and the section interior or ‘internal’ area. F4/80: pan-macrophage marker, DDX4: pan-germ cell marker, laminin: marks cord basement membrane. Insets in B and C show nuclear staining with hematoxylin and DAPI, respectively. Insets show lack of signal in negative control sections lacking primary antibodies. (JPG 516 KB)

Supplementary file2 Supplementary Figure S2. Two distinct F4/80+ cell populations at E15.5, each exhibiting a distinctive overall size, nuclear shape and F4/80 IF signal level in E15.5 mouse testis sections. A1 and B1: Small rounded F4/80Dim cells. A2: Co-localisation of two small rounded F4/80+ cells with band shaped and rounded nuclei. A3 and A4: Small F4/80+ cells with segmented and band shaped nuclei. A5, B2 and B3: Large and elongated F4/80Hi. Cells within numbered white boxes in A and B are shown in corresponding images in A.1 - A.5 and B.1 - B.3. Marker colours are indicated on image. (JPG 603 KB)

Supplementary file3 Supplementary Figure S3. A marked increase in CD3+ (T cell) abundance occurs between E15.5 and PND0 in the mouse testis. Arrows in A1 and A2 highlight the detection of a single CD3+ cell E15.5 testis sections from two individual animals. B1 and B2 illustrated detection of multiple CD3+ cells in two individual PND0 sections. CD3: pan-T cell marker, DAPI: nuclear stain. Insets at higher magnification show the size and shape of CD3+ cells. Marker colours are indicated on the figure. (JPG 821 KB)

Supplementary file4 Supplementary Figure S4. A marked redistribution of macrophages (F4/80+) from the testis perimeter to the interior occurs from E13.5 to PND0. Dotted lines designate the border of the testis with the mesonephros or epididymis regions. The rete testis is designated between two dotted lines on the E15.5 section. Marker colours are indicated on figure. (JPG 477 KB)

Supplementary file5 Supplementary Figure S5. F4/80+ cells inside E15.5 testis cords. 1, 2: adjacent to basement membrane, and 3: in the cord centre. Numbered white boxes on left hand low magnification image are enlarged on right hand side. Marker colours are indicated on image. (JPG 576 KB)

Supplementary file6 Supplementary Figure S6. F4/80+/CD206+ interactions, shape and cell distribution patterns in PND0 mouse testis sections. A: Contact between two large and elongated F4/80Hi/CD206+ cells. B: An elongated F4/80+CD206+ cell. C: Large and elongated CD206+ macrophages are the predominant macrophage phenotype in the testis section perimeter. Marker colours are indicated beside each panel set. (JPG 264 KB)

Supplementary file7 Supplementary Figure S7. F4/80+/CD206+ and F4/80-/MHC Class II+ cell distribution patterns in E15.5 and PND0 mouse testis sections. A, A′, A″: This section illustrates the significantly higher number of CD206+ macrophages in the mesonephros/epididymis compared to the testis at E15.5. B and B´: The distribution of F4/80+/CD206+ cells across the whole testis section at PND0. C1-3: A low number of small, rounded F4/80-/MHC Class II+ cells are widely distributed in the PND0 mouse testis. Each panel corresponds to an individual animal. C3. Numbered white boxes (1-3) in the low magnification image are shown at high magnification in the right-hand panels. Marker colours are indicated on the figure. (JPG 1161 KB)

Supplementary file8 Supplementary Figure S8. Distribution pattern of Ly6G+ cells. A: This E13.5 testis section displayed a single Ly6G+ cell, with a band-shaped nucleus. B1 - B3: Asymmetric distribution of neutrophils in the testis interior at E15.5; each corresponds to an individual animal. A′, B3′: The white box on the low magnification image on the left is shown on the right in higher magnification. C and C′: Ly6G+ cells were frequently detected inside cords (white rectangle) and in the interstitium at PND0. Marker colours are shown above. (JPG 948 KB)

′

Supplementary file9 (DOCX 24 KB)

Supplementary file10 (DOCX 14 KB)

## Data Availability

The data used in this study are available from the corresponding authors upon reasonable request.
