# Supplementary figures and images for: The changing landscape of immune cells in the fetal mouse testis
Source: Histochem Cell Biol. 2022 Jul 12;158(4):345–68. doi: 10.1007/s00418-022-02129-6 (PMC9512757; doi:10.1007/s00418-022-02129-6)

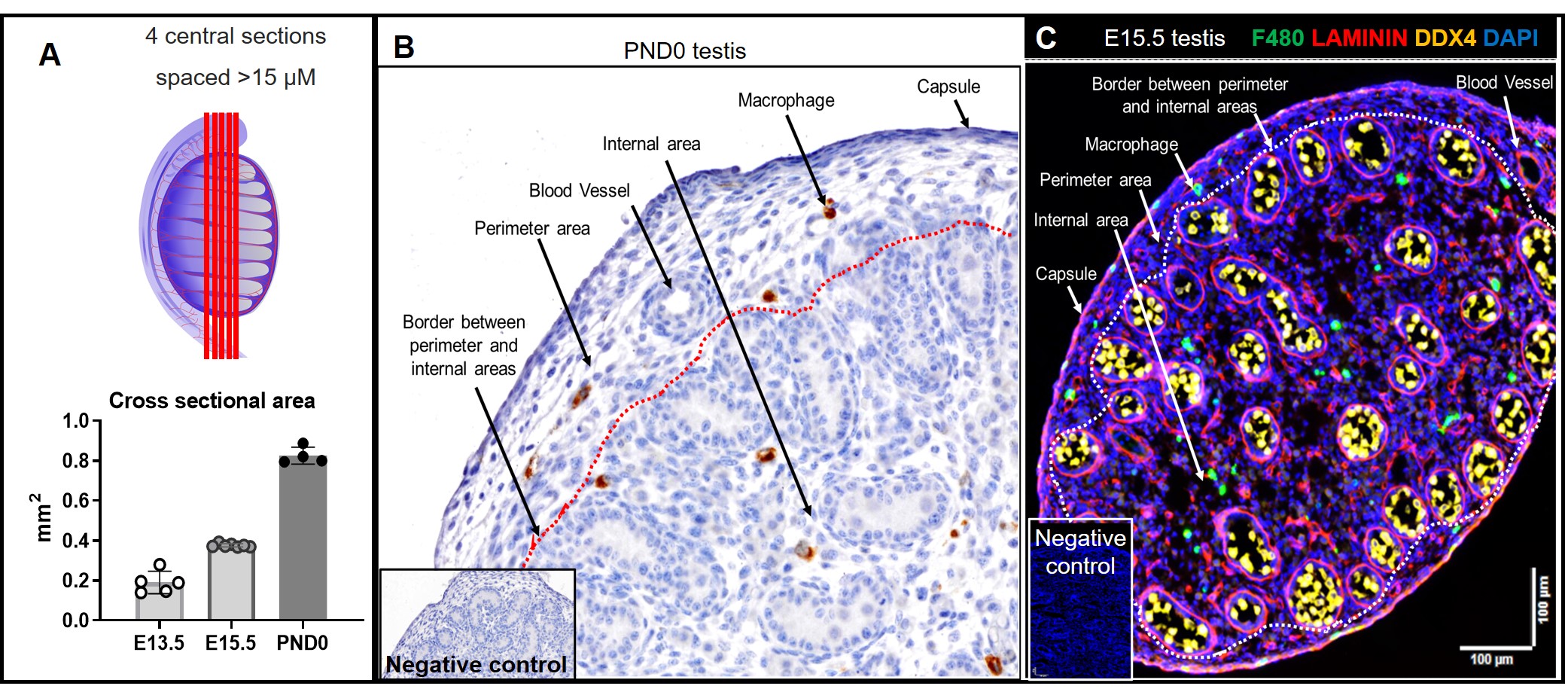

Supplement: Supplementary file 1 — Supplementary file1 Supplementary Figure S1. Histology and approach to analysis of cell populations in sections. A. Cross sectional area (mm2) of mouse testis sections at E13.5, E15.5 and PND0. Each data point represents the average cross-sectional area of 4 sections from the testis of an individual animal, shown with mean and SD. B and C. Delineation of perimeter and internal areas of fetal mouse testis cross sections. Two different locations of F4/80+ cells are shown using (B) immunohistochemistry (brown stain) in PND0 testis and (C) immunofluorescence in E15.5 testis (macrophages (green), germ cells (yellow) and cord boundary (red)). Dotted lines denote the division between the section ‘perimeter’, between the testis capsule and edge of the outermost cord, and the section interior or ‘internal’ area. F4/80: pan-macrophage marker, DDX4: pan-germ cell marker, laminin: marks cord basement membrane. Insets in B and C show nuclear staining with hematoxylin and DAPI, respectively. Insets show lack of signal in negative control sections lacking primary antibodies. (JPG 516 KB) [file 418_2022_2129_MOESM1_ESM.jpg]

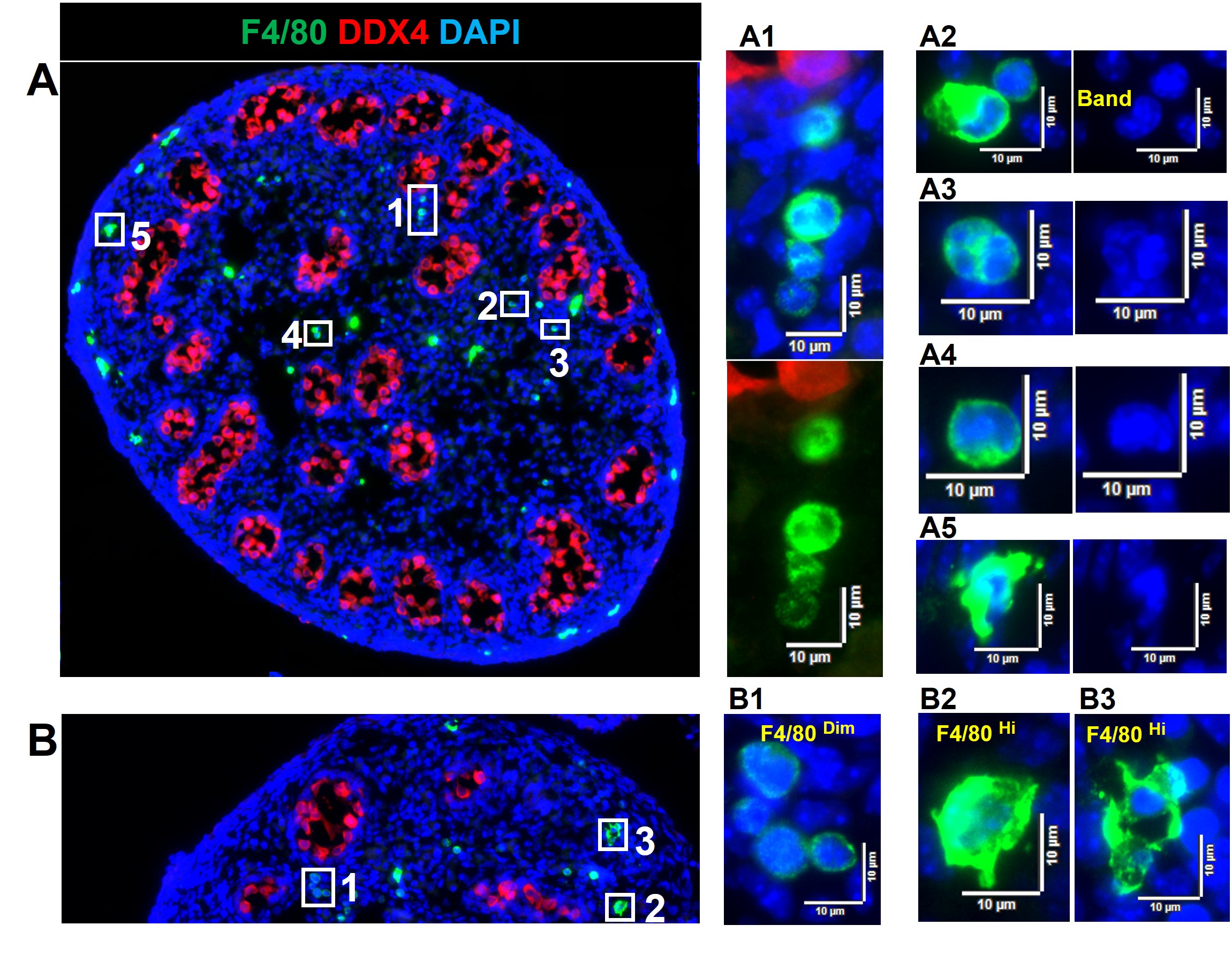

Supplement: Supplementary file 2 — Supplementary file2 Supplementary Figure S2. Two distinct F4/80+ cell populations at E15.5, each exhibiting a distinctive overall size, nuclear shape and F4/80 IF signal level in E15.5 mouse testis sections. A1 and B1: Small rounded F4/80Dim cells. A2: Co-localisation of two small rounded F4/80+ cells with band shaped and rounded nuclei. A3 and A4: Small F4/80+ cells with segmented and band shaped nuclei. A5, B2 and B3: Large and elongated F4/80Hi. Cells within numbered white boxes in A and B are shown in corresponding images in A.1 - A.5 and B.1 - B.3. Marker colours are indicated on image. (JPG 603 KB) [file 418_2022_2129_MOESM2_ESM.jpg]

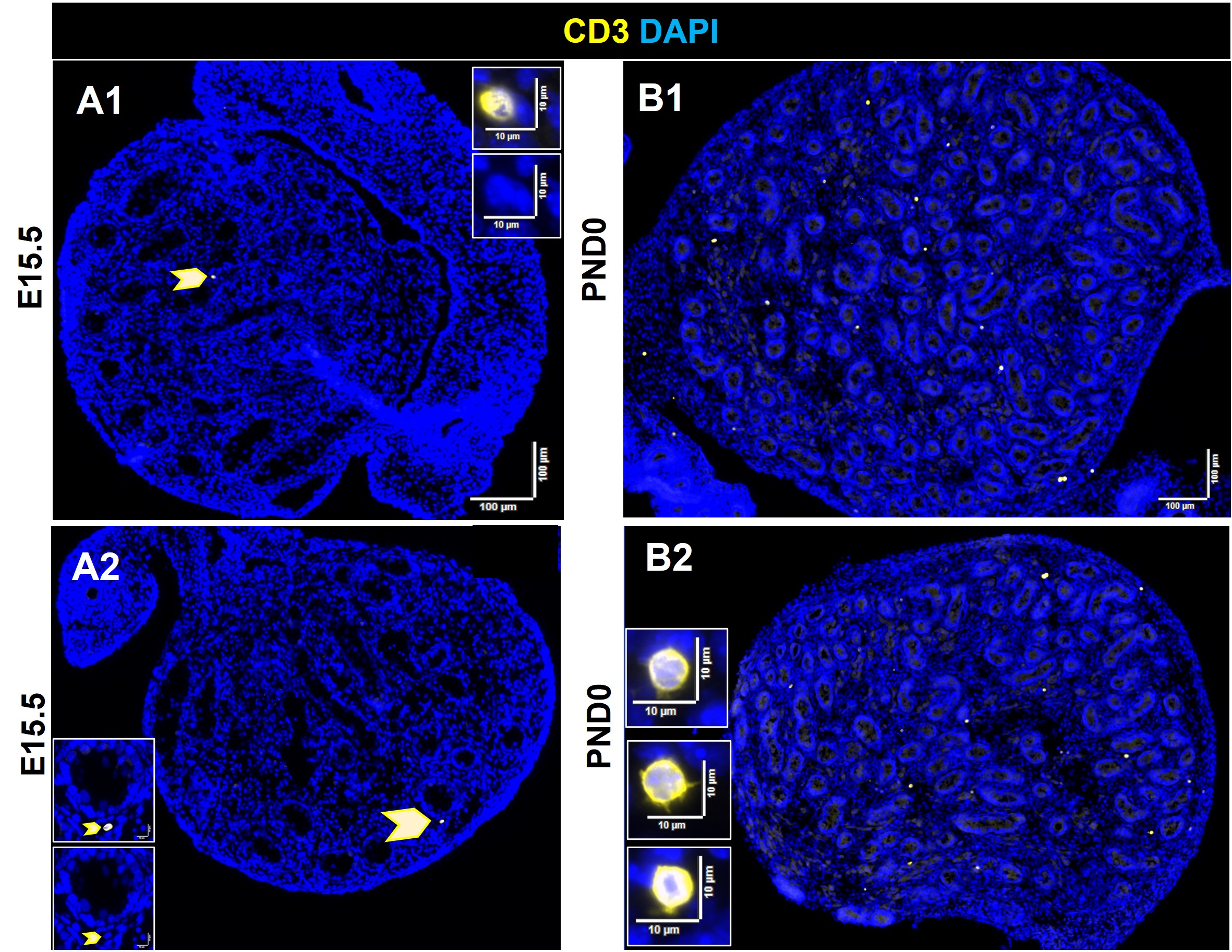

Supplement: Supplementary file 3 — Supplementary file3 Supplementary Figure S3. A marked increase in CD3+ (T cell) abundance occurs between E15.5 and PND0 in the mouse testis. Arrows in A1 and A2 highlight the detection of a single CD3+ cell E15.5 testis sections from two individual animals. B1 and B2 illustrated detection of multiple CD3+ cells in two individual PND0 sections. CD3: pan-T cell marker, DAPI: nuclear stain. Insets at higher magnification show the size and shape of CD3+ cells. Marker colours are indicated on the figure. (JPG 821 KB) [file 418_2022_2129_MOESM3_ESM.jpg]

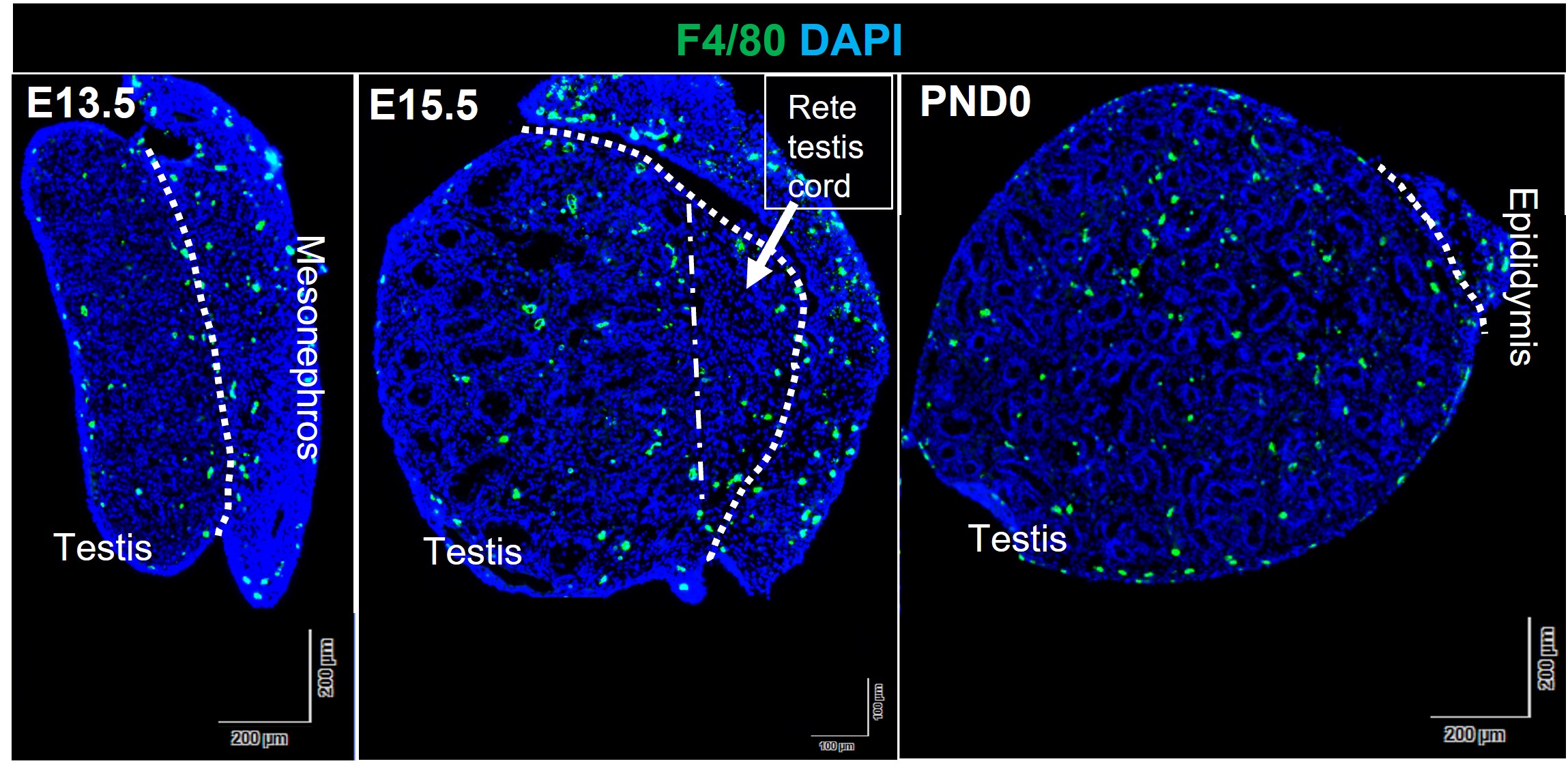

Supplement: Supplementary file 4 — Supplementary file4 Supplementary Figure S4. A marked redistribution of macrophages (F4/80+) from the testis perimeter to the interior occurs from E13.5 to PND0. Dotted lines designate the border of the testis with the mesonephros or epididymis regions. The rete testis is designated between two dotted lines on the E15.5 section. Marker colours are indicated on figure. (JPG 477 KB) [file 418_2022_2129_MOESM4_ESM.jpg]

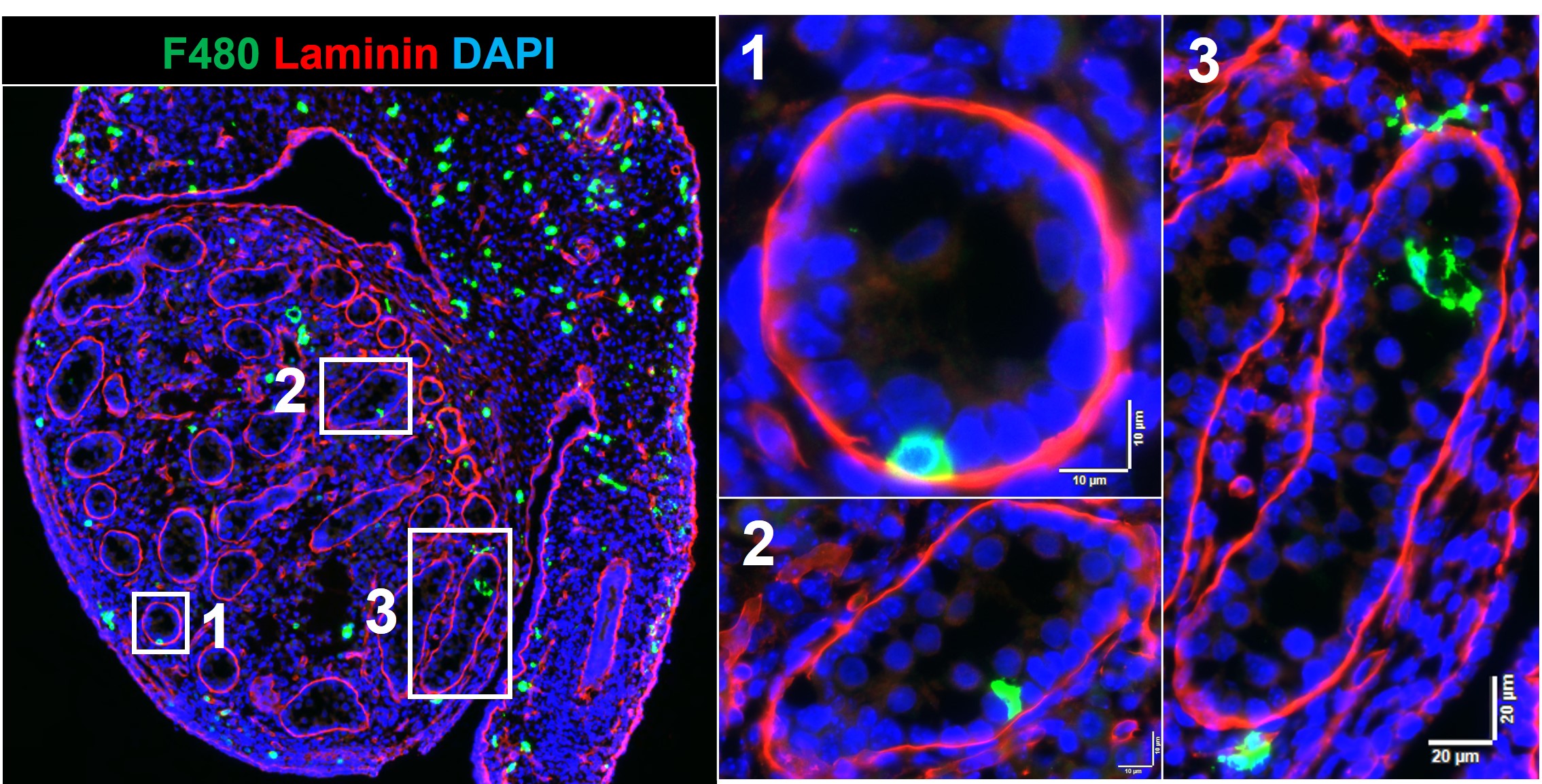

Supplement: Supplementary file 5 — Supplementary file5 Supplementary Figure S5. F4/80+ cells inside E15.5 testis cords. 1, 2: adjacent to basement membrane, and 3: in the cord centre. Numbered white boxes on left hand low magnification image are enlarged on right hand side. Marker colours are indicated on image. (JPG 576 KB) [file 418_2022_2129_MOESM5_ESM.jpg]

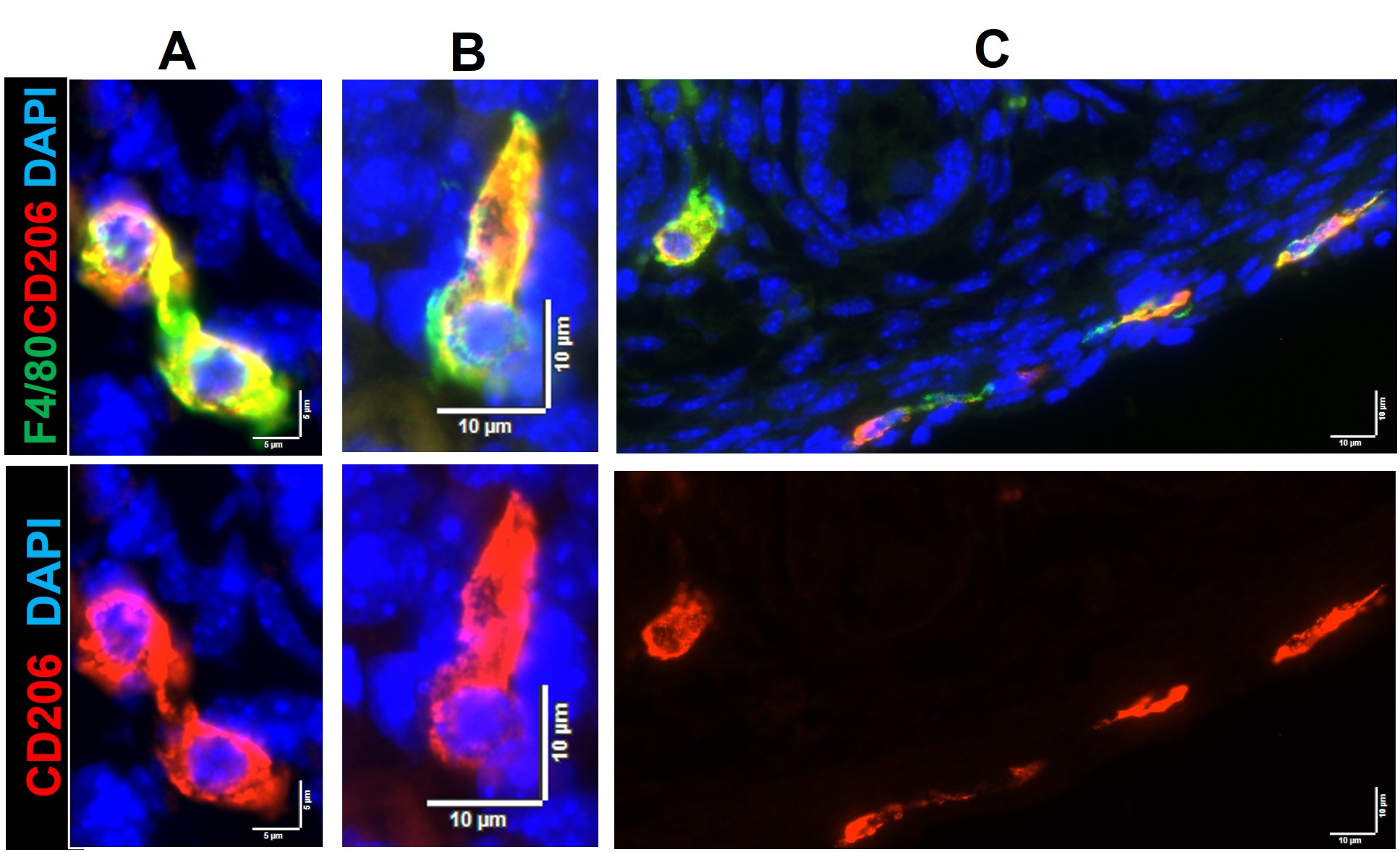

Supplement: Supplementary file 6 — Supplementary file6 Supplementary Figure S6. F4/80+/CD206+ interactions, shape and cell distribution patterns in PND0 mouse testis sections. A: Contact between two large and elongated F4/80Hi/CD206+ cells. B: An elongated F4/80+CD206+ cell. C: Large and elongated CD206+ macrophages are the predominant macrophage phenotype in the testis section perimeter. Marker colours are indicated beside each panel set. (JPG 264 KB) [file 418_2022_2129_MOESM6_ESM.jpg]

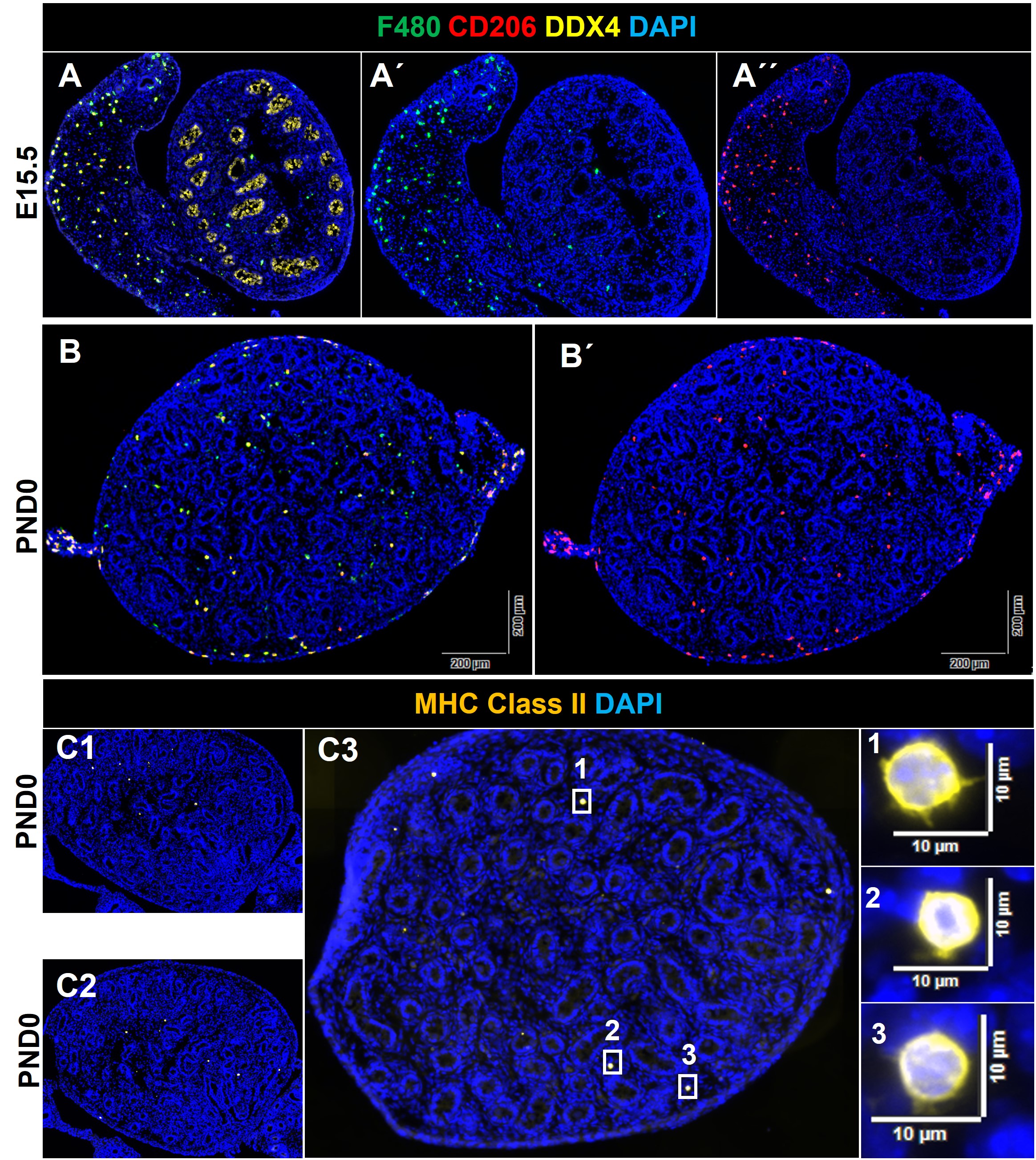

Supplement: Supplementary file 7 — Supplementary file7 Supplementary Figure S7. F4/80+/CD206+ and F4/80-/MHC Class II+ cell distribution patterns in E15.5 and PND0 mouse testis sections. A, A′, A″: This section illustrates the significantly higher number of CD206+ macrophages in the mesonephros/epididymis compared to the testis at E15.5. B and B´: The distribution of F4/80+/CD206+ cells across the whole testis section at PND0. C1-3: A low number of small, rounded F4/80-/MHC Class II+ cells are widely distributed in the PND0 mouse testis. Each panel corresponds to an individual animal. C3. Numbered white boxes (1-3) in the low magnification image are shown at high magnification in the right-hand panels. Marker colours are indicated on the figure. (JPG 1161 KB) [file 418_2022_2129_MOESM7_ESM.jpg]

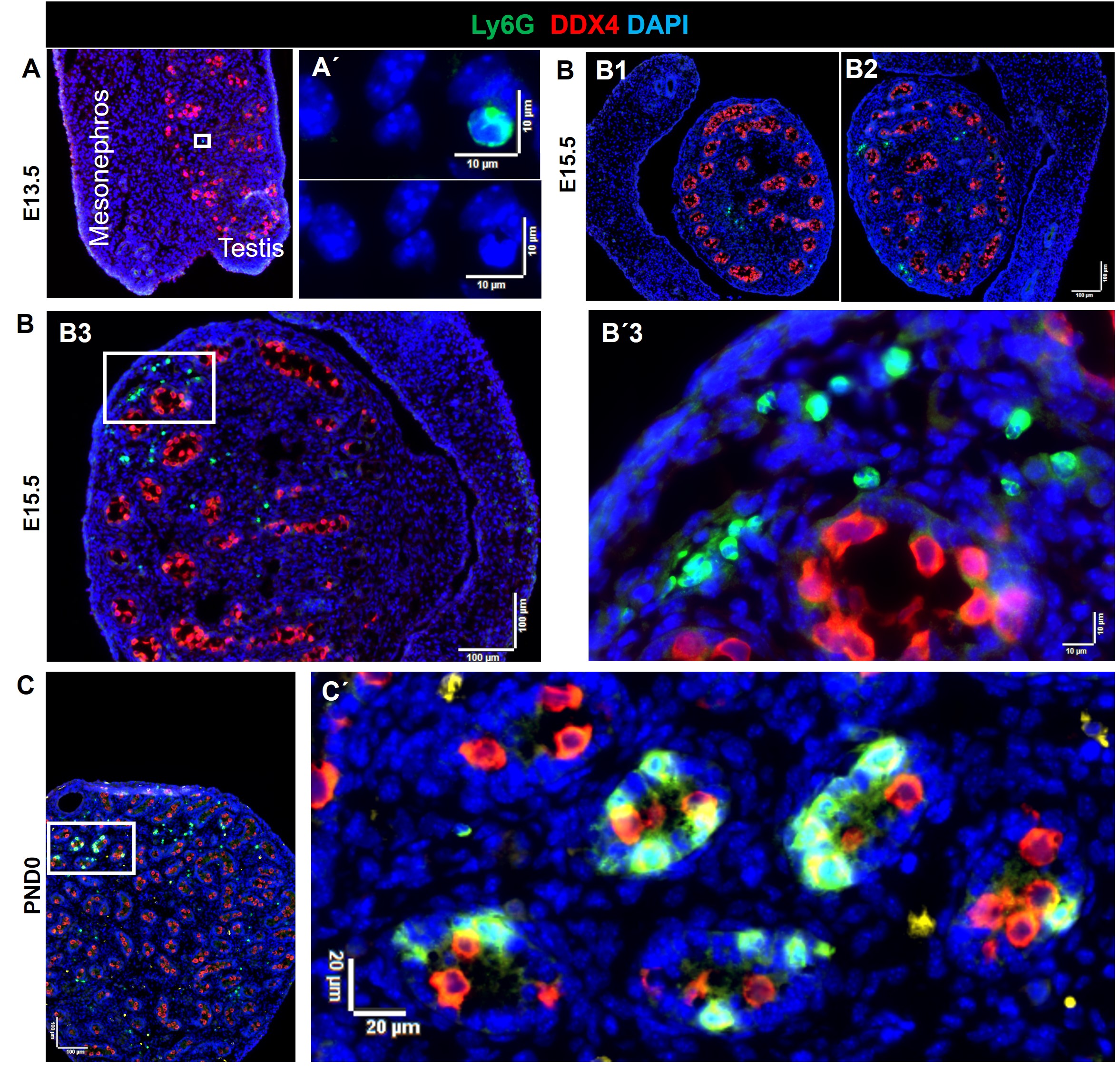

Supplement: Supplementary file 8 — Supplementary file8 Supplementary Figure S8. Distribution pattern of Ly6G+ cells. A: This E13.5 testis section displayed a single Ly6G+ cell, with a band-shaped nucleus. B1 - B3: Asymmetric distribution of neutrophils in the testis interior at E15.5; each corresponds to an individual animal. A′, B3′: The white box on the low magnification image on the left is shown on the right in higher magnification. C and C′: Ly6G+ cells were frequently detected inside cords (white rectangle) and in the interstitium at PND0. Marker colours are shown above. (JPG 948 KB) [file 418_2022_2129_MOESM8_ESM.jpg]
