# Supplementary material for: The changing landscape of immune cells in the fetal mouse testis
Source: Histochem Cell Biol. 2022 Jul 12;158(4):345–68. doi: 10.1007/s00418-022-02129-6 (PMC9512757; doi:10.1007/s00418-022-02129-6)
Supplement: Supplementary file 9 — Supplementary file9 (DOCX 24 KB) [file 418_2022_2129_MOESM9_ESM.docx]

**Table S1.** Primary and secondary antibodies used for immunohistochemistry, indirect immunofluorescence, and direct immunofluorescence.

| Antibodies | Source (citations) | Conjugation | Cat # | Clone/ Host | Dilution |
| --- | --- | --- | --- | --- | --- |
| F4/80 | Prof R. Kitching lab  (Indumathy et al., 2020; Jones et al., 2009) | - | In-house | Polyclonal/ rat | 1:200 |
| F4/80 | eBioscience  (You et al., 2008; Devey et al., 2009; Tuit et al., 2019; Indumathy et al., 2020) | APC | 17-4801-82 | BM8/ rat | 1:200 |
| CD45 | Merck Millipore  (Indumathy et al., 2020) | - | 05-1416 | IBL-5/25/ rat | 1:100 |
| Ly6G | Bio X Cell  (Woytschak et al., 2016; Adrover et al., 2019) | - | BE0075 | 1A8/ rat | 1:4000 |
| DDX4 (VASA) | R&D Systems  (Wang et al., 2017; Hermann et al., 2018; Stringer et al., 2018) | - | AF2030 | Polyclonal/ goat | 1:400 |
| Laminin | Sigma-Aldrich | - | L9393 | Polyclonal/ rabbit | 1:1000 |
| Biotinylated Rabbit anti-rat | DAKO | - | E0468 | Polyclonal/ rabbit | 1:500 |
| Biotinylated Rabbit anti- goat | DAKO | - | E0466 | Polyclonal/ rabbit | 1:500 |
| CD45 | BD Pharmingen  (Zuba-Surma et al., 2008; Rai et al., 2020) | APC | 559864 | 30-F11/ rat | 1:100 |
| MHC Class II  (I-A/I-E) | BioLegend  (Hamaidi et al., 2020; Stephens et al., 2021) | Alexa Fluor 647 | 107618 | M5/114.15.2/ rat | 1:400 |
| CD206 | BioLegend  (Linde et al., 2018; Boonchan et al., 2021) | Alexa Fluor 594 | 141726 | C068C2/ rat | 1:500 |
| CD3 | BD Pharmingen  (Breitbach et al., 2018; Indumathy et al., 2020) | Alexa Fluor 647 | 557869 | 17A2/ rat | 1:100 |
| Donkey anti-rat | Invitrogen | Alexa Fluor 488 | A21208 | Donkey | 1:500 |
| Donkey anti-rabbit | Invitrogen | Alexa Fluor 555 | A31572 | Donkey | 1:500 |
| Rabbit anti-goat | Life Technologies | Alexa Fluor 456 | A21085 | Rabbit | 1:500 |
| Donkey anti-goat | Life Technologies | Alexa Fluor 647 | A21447 | Donkey | 1:500 |
| Donkey anti-rabbit | Life Technologies | Alexa Fluor 647 | A31573 | Donkey | 1:500 |

**References:**

Adrover JM, Del Fresno C, Crainiciuc G, Cuartero MI, Casanova-Acebes M, Weiss LA, et al. (2019) A neutrophil timer coordinates immune defense and vascular protection. *Immunity.* 390-402.e10. doi:10.1016/j.immuni.2019.01.002

Averill MM, Kerkhoff C, Bornfeldt KE. (2012) S100A8 and S100A9 in cardiovascular biology and disease. *Arterioscler Thromb Vasc*. 32(2):223-9. doi: 10.1161/ATVBAHA.111.236927.

Boonchan M, Arimochi H, Otsuka K, Kobayashi T, Uehara H, Jaroonwitchawan T. et al. (2021) Necroptosis protects against exacerbation of acute pancreatitis. *Cell Death Dis* 10;12(6):601. doi: 10.1038/s41419-021-03847-w.

Breitbach M, Kimura K, Luis TC, Fuegemann CJ, Woll PS, Hesse M, et al. (2018) In Vivo Labeling by CD73 Marks Multipotent Stromal Cells and Highlights Endothelial Heterogeneity in the Bone Marrow Niche. *Cell Stem Cell* 1;22(2):262-276.e7. doi: 10.1016/j.stem.2018.01.008.

Devey L, Ferenbach D, Mohr E, Sangster K, Bellamy CO, Hughes J, et al. (2009) Tissue-resident macrophages protect the liver from ischemia reperfusion injury via a heme oxygenase-1-dependent mechanism. *Mol Ther* 17(1):65-72. doi: 10.1038/mt.2008.237.

Hamaidi I, Zhang L, Kim N, Wang MH, Iclozan C, Fang B, et al. (2020) Sirt2 Inhibition Enhances Metabolic Fitness and Effector Functions of Tumor-Reactive T Cells. *Cell Metab* 1;32(3):420-436.e12. doi: 10.1016/j.cmet.2020.07.008.

Hermann BP, Cheng K, Singh A, Roa-De La Cruz L, Mutoji KN, Chen IC, et al. (2018) The Mammalian Spermatogenesis Single-Cell Transcriptome, from Spermatogonial Stem Cells to Spermatids. *Cell Rep* 6;25(6):1650-1667.e8. doi: 10.1016/j.celrep.2018.10.026.

Hobbs JA, May R, Tanousis K, McNeill E, Mathies M, Gebhardt C, et al. (2003) Myeloid cell function in MRP-14 (S100A9) null mice. *Mol Cell Biol* 23(7):2564-2576. doi:10.1128/MCB.23.7.2564-2576.2003

Indumathy S, Pueschl D, Klein B, Fietz D, Bergmann M, Schuppe HC, et al. (2020) Testicular immune cell populations and macrophage polarisation in adult male mice and the influence of altered activin A levels. *J Reprod Immunol* 142:103204. doi: 10.1016/j.jri.2020.103204.

Jones LK, O'Sullivan KM, Semple T, Kuligowski MP, Fukami K, Ma FY, et al. (2009) IL-1RI deficiency ameliorates early experimental renal interstitial fibrosis. *Nephrol Dial Transplant* 24(10):3024-32. doi: 10.1093/ndt/gfp214.

Linde N, Casanova-Acebes M, Sosa MS, Mortha A, Rahman A, Farias E, et al. (2018) Macrophages orchestrate breast cancer early dissemination and metastasis. *Nat Commun* 2;9(1):21. doi: 10.1038/s41467-017-02481-5.

Pepper RJ, Wang HH, Rajakaruna GK, Papakrivopoulou E, Vogl T, Pusey CD, et al. (2015) S100A8/A9 (calprotectin) is critical for development of glomerulonephritis and promotes inflammatory leukocyte-renal cell interactions. *Am J Pathol*185(5):1264-74. doi: 10.1016/j.ajpath.2015.01.015.

Rai V, Wood MB, Feng H, Schabla NM, Tu S, Zuo J. (2020) The immune response after noise damage in the cochlea is characterized by a heterogeneous mix of adaptive and innate immune cells. *Sci Rep* 16;10(1):15167. doi: 10.1038/s41598-020-72181-6.

Stephens WZ, Kubinak JL, Ghazaryan A, Bauer KM, Bell R, Buhrke K, et al. (2021) Epithelial-myeloid exchange of MHC class II constrains immunity and microbiota composition. *Cell Rep* 2;37(5):109916. doi: 10.1016/j.celrep.2021.109916.

Stringer JM, Forster SC, Qu Z, Prokopuk L, O'Bryan MK, Gardner DK, et al. Reduced PRC2 function alters male germline epigenetic programming and paternal inheritance. *BMC Biol* (2018) 20;16(1):104. doi: 10.1186/s12915-018-0569-5.

Tuit S, Salvagno C, Kapellos TS, Hau CS, Seep L, Oestreich M, et al. (2019) Transcriptional signature derived from murine tumor-associated macrophages correlates with poor outcome in breast cancer patients. *Cell Rep* 29;29(5):1221-1235.e5. doi: 10.1016/j.celrep.2019.09.067.

Vogl T, Ludwig S, Goebeler M, Strey A, Thorey IS, Reichelt R, et al. (2004) MRP8 and MRP14 control microtubule reorganization during transendothelial migration of phagocytes. *Blood* 15;104(13):4260-8. doi: 10.1182/blood-2004-02-0446.

Wang N, Satirapod C, Ohguchi Y, Park ES, Woods DC, Tilly JL. (2017) Genetic studies in mice directly link oocytes produced during adulthood to ovarian function and natural fertility. *Sci Rep* 30;7(1):10011. doi: 10.1038/s41598-017-10033-6.

Wang S, Song R, Wang Z, Jing Z, Wang S, Ma J. (2018) S100A8/A9 in Inflammation. *Front Immunol* 11; 9:1298. doi: 10.3389/fimmu.2018.01298.

Woytschak J, Keller N, Krieg C, Impellizzieri D, Thompson RW, Wynn TA, et al. (2016) Type 2 interleukin-4 receptor signaling in neutrophils antagonizes their expansion and migration during infection and inflammation. *Immunity* 19;45(1):172-84. doi: 10.1016/j.immuni.2016.06.025.

You Q, Cheng L, Kedl RM, Ju C. (2008) Mechanism of T cell tolerance induction by murine hepatic Kupffer cells. *Hepatology* 48(3):978-90. doi: 10.1002/hep.22395.

Zuba-Surma EK, Kucia M, Dawn B, Guo Y, Ratajczak MZ, Bolli R. (2008) Bone marrow-derived pluripotent very small embryonic-like stem cells (VSELs) are mobilized after acute myocardial infarction. *J Mol Cell Cardiol* 44(5):865-73. doi: 10.1016/j.yjmcc.2008.02.279.
