# Supplementary material for: The changing landscape of immune cells in the fetal mouse testis
Source: Histochem Cell Biol. 2022 Jul 12;158(4):345–68. doi: 10.1007/s00418-022-02129-6 (PMC9512757; doi:10.1007/s00418-022-02129-6)
Supplement: Supplementary file 10 — Supplementary file10 (DOCX 14 KB) [file 418_2022_2129_MOESM10_ESM.docx]

**Table S2.** Primer pairs used for qRT-PCR.

| **Gene** | **Forward (5'-3')** | **Reverse (5'-3')** | **Gene ID** |
| --- | --- | --- | --- |
| *Rplp0* | GGACCCGAGAAGACCTCCTT | GCACATCACTCAGAATTTCAATGG | NM_007475.5 |
| *F4/80* | AGCACCATGTTAGCTGCTCT | GGGGCCCCTGTAGATACTGA | NM_010130.4 |
| *Cd45* | ATGGTCCTCTGAATAAAGCCCA | TCAGCACTATTGGTAGGCTCC | NM_011661.5 |
| *Cd3* | ATGCGGTGGAACACTTTCTGG | GCACGTCAACTCTACACTGGT | NM_007648.4 |
| *Cd206* | TTTCGGTGGACTGTGGACGA | ATAAGCCACCTGCCACTCCG | NM_008625.2 |
| *Ly6g* | TTGCAAAGTCCTGTGTGCTC | AGGGGCAGGTAGTTGTGTTG | NM_001310438.1 (Li et al., 2015) |
| *Mhc class ii*  *(H2-ab1)* | TGGCCTTTTCATCCGTCACA | ACTGGCAGTCAGGAATTCGG | NM_207105.3 |
| *Ddx4* | CATCGAATTGGACGCACTG | GGCAATCTCTTCTAGCCATGC | NM_001145885.1 |

**Reference**

Li Y, Qi X, Liu B, Huang H. (2015) The STAT5-GATA2 pathway is critical in basophil and mast cell differentiation and maintenance. *J Immunol*. 1;194(9):4328-38. doi: 10.4049/jimmunol.1500018.
